# Exploration of Dark Chemical Genomics Space via Portal Learning: Applied to Targeting the Undruggable Genome and COVID-19 Anti-Infective Polypharmacology

**DOI:** 10.21203/rs.3.rs-1109318/v1

**Published:** 2021-12-01

**Authors:** Tian Cai, Li Xie, Muge Chen, Yang Liu, Di He, Shuo Zhang, Cameron Mura, Philip E. Bourne, Lei Xie

**Affiliations:** 1 Ph.D. Program in Computer Science, The Graduate Center, The City University of New York, New York, 10016, USA; 2 Department of Computer Science, Hunter College, The City University of New York, New York, 10065, USA; 3 Master Program in Computer Science, Courant Institute of Mathematical Sciences, New York University; 4 School of Data Science & Department of Biomedical Engineering, University of Virginia, Virginia, 22903, USA; 5 Helen and Robert Appel Alzheimer’s Disease Research Institute, Feil Family Brain & Mind Research Institute, Weill Cornell Medicine, Cornell University, New York, 10021, USA

**Keywords:** biomedicine, portal learning, machine learning, COVID-19, SARS-CoV-2

## Abstract

Advances in biomedicine are largely fueled by exploring uncharted territories of human biology. Machine learning can both enable and accelerate discovery, but faces a fundamental hurdle when applied to unseen data with distributions that differ from previously observed ones—a common dilemma in scientific inquiry. We have developed a new deep learning framework, called *Portal Learning*, to explore dark chemical and biological space. Three key, novel components of our approach include: (i) end-to-end, step-wise transfer learning, in recognition of biology’s sequence-structure-function paradigm, (ii) out-of-cluster meta-learning, and (iii) stress model selection. Portal Learning provides a practical solution to the out-of-distribution (OOD) problem in statistical machine learning. Here, we have implemented Portal Learning to predict chemical-protein interactions on a genome-wide scale. Systematic studies demonstrate that Portal Learning can effectively assign ligands to unexplored gene families (unknown functions), versus existing state-of-the-art methods. Compared with AlphaFold2-based protein-ligand docking, Portal Learning significantly improved the performance by 79% in PR-AUC and 27% in ROC-AUC, respectively. The superior performance of Portal Learning allowed us to target previously “undruggable” proteins and design novel polypharmacological agents for disrupting interactions between SARS-CoV-2 and human proteins. Portal Learning is general-purpose and can be further applied to other areas of scientific inquiry.

## 1 Introduction

The central aim of scientific inquiry has been to deduce new concepts from existing knowledge or generalized observations. The biological sciences offer numerous such challenges. The rise of deep learning has spurred major interest in using machine learning to explore uncharted molecular and functional spaces in biology and medicine, ranging from ‘deorphanizing’ G-protein coupled receptors[1] and translating cell-line screens to patient drug responses[2][3], to predicting novel protein structures[4][5][6], to identifying new cell types from single-cell omics data[7]. Illuminating the dark space of human knowledge is a fundamental problem that one can attempt to address via deep learning—that is, to generalize a “well-trained” model to unseen data that lies out-of-the-distribution (OOD) of the training data, in order to successfully predict outcomes from conditions that the model has never before encountered. While deep learning is capable, in theory, of simulating any functional mapping, its generalization power is notoriously limited in the case of distribution shifts[8].

The training of a deep learning model starts with a domain-specific model architecture. The final model instance that is selected, and its performance, are determined by a series of data-dependent design choices, including model initialization, data used for training, validation, and testing, optimization of loss function, and evaluation metrics. Each of these design choices impacts the generalization power of a trained model. The development of several recent deep learning-based approaches—notably transfer learning[9], self-supervised representation learning[10], and meta-learning [11][12]—has been motivated by the OOD challenge. However, each of these methods focuses on only one aspect in the training pipeline of a deep model. Causal learning and mechanism-based modeling could be a more effective solution to the OOD problem [8], but at present these approaches can be applied only on modest scales because of data scarcity and limited domain knowledge. Solving large-scale OOD problems in biomedicine, via machine learning, would benefit from a systematic framework for integrative, beginning-to-end model development, training, and testing.

Here, we propose a new deep learning framework, called *Portal Learning*, that systematically addresses the three OOD vulnerabilities in a training pipeline: specifically, we employ biology-inspired model initialization, optimization on an OOD loss, and model selection methods. We define ‘*portal*’ as a model with an initialized instance that is (preferably) close to the global optimum in some learning ‘*universe*’. The *universe* includes a specific input data-set, specific tasks, and a model architecture that provides a functional mapping from the data-set (and associated distributions) to the tasks. Note that, even with the same model architecture, changes in a pipeline’s associated data-set correspond to changes in the universe. Portal Learning takes a global view to design training schemes that are task-specific and use domain knowledge as constraints to guide the exploration of the learning space.

To assess the utility of Portal Learning, we implemented this concept as a concrete framework, termed *PortalCG*, for predicting small-molecule binding to dark gene families (i.e., those with no annotated ligands). Despite tremendous progress in high-throughput screening, the majority of chemical genomics space remains unexplored or ‘dark’ [13] (more details in results). Elucidating dark gene families can illuminate many fundamental but only poorly characterized biological pathways, such as microbiome-host interactions mediated by metabolite-protein interactions. Such efforts could also provide novel approaches for identifying new druggable targets and discovering effective therapeutic strategies for currently incurable diseases; for instance, in Alzheimer’s disease (AD) many disease-associated genes have been identified from multiple omics studies, but are currently considered un-druggable [14]. Accurately predicting chemical-protein interactions (CPIs) on a genome-wide scale is a challenging OOD problem[1]. If one considers only the reported area under the receiver operating characteristic curve (AUROC), which has achieved 0.9 in many state-of-the-art methods[15][16], it may seem the problem has been solved. However, the performance has been primarily measured in scenarios where the data distribution in the test set does not differ significantly from that in the training set, in terms of identities of proteins or types of chemicals. Few sequence-based methods have been developed and evaluated for an out-of-gene family scenario, where proteins in the test set belong to different (non-homologous) gene families than in the training set; this sampling bias is even more severe in considering cases where the new gene family does not have any reliable three-dimensional (3D) structural information. Therefore, one can fairly claim that all existing work has been confined to just narrow regions of chemical genomics space, without validated generalizability into the dark genome.

Rigorous benchmarking studies, reported herein, show that PortalCG significantly outperforms the leading methods that are available for predicting ligand binding to (dark) proteins. We applied PortalCG to predict candidate drug compounds for undrugged disease genes in the dark human genome, and we prioritized hundreds of undrugged genes that can be efficaciously targeted by existing drugs (notably, many of which involve alternative splicing and transcription factor). These novel genes and their lead compounds provide new opportunities for drug discovery. Furthermore, using PortalCG, we identified polypharmacological agents that might leverage novel drug targets in order to disrupt interactions between SARS-CoV-2 and human proteins. The rapid emergence of SARS-CoV-2 variants has posed a significant challenge to existing vaccine and anti-viral development paradigms. Gordon et al. experimentally identified 332 human proteins that interact with the SARS-CoV-2 virus[17]. This PPI map provides unique opportunities for anti-SARS-CoV-2 drug discovery: targeting the host proteins involved in PPIs can disrupt human SARS-CoV-2 interactions, thereby thwarting the onset of COVID-19. By not aiming to directly kill virions, this indirect strategy should lessen the selection pressure on viral genome evolution. A polypharmacological agent that interacts moderately strongly with multiple human proteins could be a potentially quite effective and safe anti-COVID-19 therapeutic: on the one hand, the normal functions of human proteins should not be significantly perturbed while, on the other hand, the interactions required for successful SARS-CoV-2 infection would be inhibited. Here, we virtually screened compounds in the Drug Repurposing Hub[18] against the 332 human SARS-CoV-2 interactors. Two drugs, Fenebrutinib and NMS-P715, ranked highly; interestingly, both of these anti-tumorigenic compounds inhibit kinases. Their interactions with putative human targets were supported by further (structure-based) analyses of protein-ligand binding poses.

In summary, the contributions of this work are three-fold:

1. A novel, generalized training scheme, *Portal Learning*, is proposed as a way to guide biology-inspired systematic design in order to improve the generalization power of machine learning on OOD problems, such as is found in the dark regions of molecular/functional space.
2. To concretely illustrate the Portal Learning approach, a specific algorithm, PortalCG, is proposed and implemented. Comprehensive benchmark studies demonstrate the promise of PortalCG when applied to OOD problems, specifically for exploring the dark regions of chemical genomics space.
3. Using PortalCG, we shed new light on unknown protein functions in dark genomes (viz. small molecule-binding properties), and open new avenues in polypharmacology and drug repurposing; as demonstrated by identifying novel drug targets and lead compounds for AD and anti-SARS-CoV-2 polypharmacology.

## 2 Conceptual basis of Portal Learning

**Figure 1:**
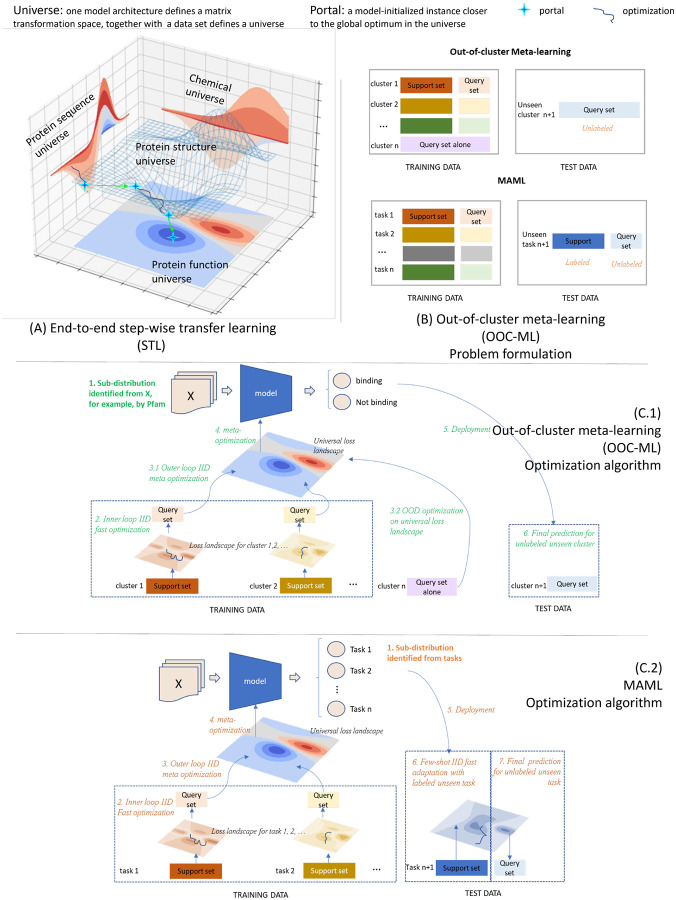
Illustration of two of the three major Portal Learning components for OOD problems, End-to-end step-wise transfer learning (STL) and out-of-cluster meta-learning (OOC-ML), using the prediction of out-of-gene family chemical-protein interactions (CPIs) as an example: **A. STL**: 3D structure of protein ligand binding site is in the center connecting protein sequences to CPIs. There are two portals, the first traveling from the protein sequence universe to the binding site structure universe by pre-training a protein language model that is optimal in the protein sequence universe and leads to a model initialization instance closer to the global optimum in the binding site structure universe. The optimization based on this initialized instance leads to the discovery of the second portal through which protein function universe gets a model initialization instance closer to its own global optimum. **B. Problem formulation of OOC-ML in comparison with MAML**: Different from MAML where training data is grouped based on the task, the training data in OOC-ML is clustered in the instance space. Instead of decomposing the data in all clusters into support and query set like MAML, there is only a query set in certain training clusters and all testing clusters in OOC-ML to simulate OOD scenario. **C. Optimization of OOC-ML in comparison with MAML**: Intuitively, OOC-ML first performs local optimizations on each cluster of training data with the support/query decomposition, then meta optimizations on the training set that has only query sets by ensembling the knowledge learned from the local optimization. The optimized model is applied to the test data in a zero-shot learning setting. In contrast, the meta-optimization in MAML requires query sets in the setting of few-shot learning.

To enable the exploration of dark regions of chemical and biological space, Portal Learning rests upon a systematic, well-principled training strategy, the underpinnings of which are shown in Figure 1. In Portal Learning, a model architecture together with a data set and a task defines a **universe**. Each universe has some global optimum with respect to the task based on a pre-defined loss function. The model-initialized instance in a universe—which could be a local optimum in the current universe, but which facilitates moving the model to the global optimum in the ultimately targeted universe—is called a **portal**. The portal is similar to a catalyst that lows the energy barrier via a transition state for a chemical reaction to occur. The dark chemical genomics space cannot be explored effectively if the learning process is confined only to the observed universe of protein sequences that have known ligands, as the known data are highly sparse and biased (details in Result section). Hence, it is critical to successfully identify portals into the dark chemical genomics universe starting from the observed protein sequence and structure universe. For clarity and ease of reference, key terms related to Portal Learning are given in the Supplemental Materials.

The remainder of this section describes the three key components of the Portal Learning approach—namely, end-to-end step-wise transfer learning (STL), out-of-cluster meta-learning (OOC-ML), and stress model selection.

### End-to-end step-wise transfer learning (STL).

Information flow in biological systems generally involves multiple intermediate steps, from a source instance to a target. For example, a discrete genotype (source) ultimately yields a downstream phenotype (target) via many steps of gene expression, in some environmental context. For predicting genotype-phenotype associations, explicit machine learning models that represent information transmission from DNA to RNA to cellular phenotype are more powerful than those that ignore the intermediate steps [19]. In Portal Learning, transcriptomics profiles can be used as a portal to link the source genetic variation (e.g., variants, SNPs, homologs, etc.) and target cellular phenotype (e.g., drug sensitivity). Using deep neural networks, this process can be modeled in an end-to-end fashion.

### Out-of-cluster meta-learning (OOC-ML).

Even if we can successfully transfer the information needed for the target through intermediate portals from the source universe, we still need additional portals to reach those many sparsely-populated regions of the dark universe that lack labeled data in the target. Inspired by Model Agnostic Meta-Learning (MAML)[11], we designed a new OOC-ML approach to explore the dark biological space. MAML cannot be directly applied to Portal Learning in the context of the OOD problem because it is designed for few-shot learning under a multi-task formulation. Few-shot learning expects to have a few labeled samples from the test data set to update the trained model during inference for a new task. This approach cannot be directly applied to predicting gene functions of dark gene families where the task (e.g., binary classification of ligand binding) is unchanged, but rather there are no labeled data for a unseen distribution that may differ significantly from the training data. In a sense, rather than MAML’s “few-shot/multi-task” problem context, mapping dark chemical/biological space is more of a “zero-shot/single-task” learning problem. A key insight of OOC-ML is to define sub-distributions (clusters) for the labeled data in the source instance universe. An example demonstrated in this paper is to define sub-distributions using Pfam families when the source instance is a protein sequence. Intuitively, OOC-ML involves a two-stage learning process. In the first stage, a model is trained using each individual labeled cluster (e.g., a given Pfam ID), thereby learning whatever knowledge is (implicitly) specific to each cluster. In the second stage, all trained models from the first stage are combined and a new ensemble model is trained, using labeled clusters that were not used in the first stage. In this way, we may extract common intrinsic patterns shared by all clusters and apply the learned essential knowledge to dark ones.

### Stress model selection.

Finally, training should be stopped at a suitable point in order to avoid overfitting. This was achieved by stress model selection. Stress model selection is designed to basically recapitulate an OOD scenario by splitting the data into OOD train, OOD development, and OOD test sets as listed in Table 1; in this procedure, the data distribution for the development set differs from that of the training data, and the distribution of the test data set differs from both the training and development data.

For additional details and perspective, the conceptual and theoretical basis of Portal Learning is further described in the Methods section of the Supplemental Materials.

## 3 Results and Discussion

### 3.1 Overview of PortalCG

**Figure 2:**
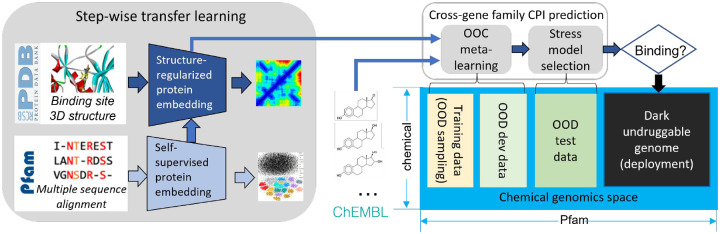
Scheme of PortalCG. PortalGC enables to predict chemical protein interactions (CPIs) for dark genes across gene families. It includes three key components: end-to-end transfer learning following sequence-structure-function paradigm, Out-of-cluster (OOC) meta-learning, and stress model selection.

We implemented the Portal Learning concept as a concrete model, PortalCG, for exploring the dark chemical genomics space. In terms of Portal Learning’s three key components (STL, OOC-ML, and stress model selection), PortalCG makes the following design choices (see also Figure 2).

#### End-to-end sequence-structure-function STL.

The function of a protein—e.g., serving as a target receptor for ligand binding—stems from its three-dimensional (3D) shape and dynamics which, in turn, is ultimately encoded in its primary amino acid sequence. In general, information about a protein’s structure is more powerful than purely sequence-based information for predicting its molecular function because sequences drift/diverge far more rapidly than do 3D structures on evolutionary timescales. Although the number of experimentally-determined structures continues to exponentially increase, and now AlphaFold2 can reliably predict 3D structures of most single-domain proteins, it nevertheless remains quite challenging to directly use protein structures as input for predicting ligand-binding properties of dark proteins. In PortalCG, protein structure information is used as a portal to connect a source protein sequence and a corresponding target protein function (Figure 1A). We begin by performing self-supervised training to map tens of millions of sequences into a universal embedding space, using our recent *distilled sequence alignment embedding* (DISAE) algorithm [1]. Then, 3D structural information about the ligand-binding site is used to fine-tune the sequence embedding. Finally, this structure-regularized protein embedding was used as a hidden layer for supervised learning of cross-gene family CPIs, following an end-to-end sequence-structure-function training process. By encapsulating the role of structure in this way, inaccuracies and uncertainties in structure prediction are ‘insulated’ and will not propagate to the function prediction.

#### Out-of-cluster meta-learning.

In the OOC-ML framework, Pfam gene families provide natural clusters as sub-distributions. In each Pfam family, the data is split into support set and query set as shown in Figure 1(B). Specifically, a model is trained for a single Pfam family independently to reach a local minimum using the support set of the Pfam family as shown in the inner loop IID optimization in Figure 1(C.1). Then a query set from the same Pfam family is used on the locally optimized model to get a loss from the local loss landscape, i.e. outer loop IID meta optimization in Figure 1(C.1). Local losses from the query sets of multiple Pfam families will be aggregated to calculate the loss on a global loss landscape, i.e. meta optimization in Figure 1(C.1). For some cluster with very limited number of data, they don’t have a support set hence will only participate in the optimization on the global loss landscape. There could be many choices of aggregations. A simple way is to calculate the average loss. The aggregated loss will be used to optimize the model on the global loss landscape. Note that weights learned on each local loss landscape will be memorized during the global optimization. In our implementation, it is realized by creating a copy of the model trained from the each family’s local optimization. In this way, the local knowledge learned is ensured to be only passed to the global loss landscape by the query set loss.

#### Stress model selection.

The final model was selected using Pfam families that were not used in the training stage (Figure 2, right panel).

The Supplemental Materials provide further methodological details, covering data pre-processing, the core algorithm, model configuration, and implementation details.

### 3.2 There are significantly unexplored dark spaces in chemical genomics

**Figure 3:**
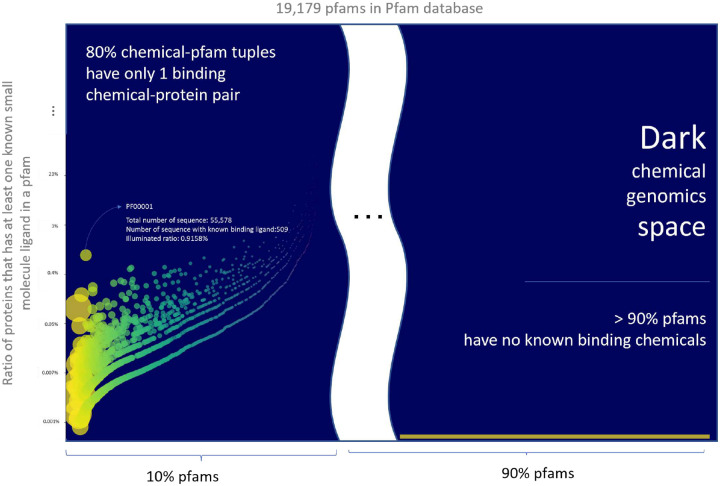
Chemical genomics space in statistics: The ratio of proteins that have at least a known ligand in each Pfam family. Each color bubble represents a Pfam family. The size of a bubble is proportional to the total number of proteins in the Pfam family. Y-axis is the ratio of proteins with known ligand(s) in a Pfam family. Around 2, 000 Pfam families have at least one known small molecule ligand. Most of these Pfam families have less than 1% proteins with known ligands. Furthermore, around 90% of total 19, 179 Pfam families are in the dark chemical genomics space without any known ligand information.

We inspected the known CPIs between (i) molecules in the manually-curated ChEMBL database, which consists of only a small portion of all chemical space, and (ii) proteins annotated in Pfam-A [20], which represents only a narrow slice of the whole protein sequence universe. The ChEMBL26[21] database supplies 1,950,765 chemicals paired to 13,377 protein targets, constituting 15,996,368 known interaction pairs. Even for just this small portion of chemical genomics space, unexplored CPIs are enormous, can be seen in the dark region in Figure 3. Approximately 90% of Pfam-A families do not have any known small-molecule binder. Even in Pfam families with annotated CPIs (e.g., GPCRs), there exists a significant number of ‘orphan’ receptors with unknown cognate ligands (Figure 3). Fewer than 1% of chemicals bind to more than two proteins, and < 0.4% of chemicals bind to more than five proteins, as shown in Supplemental Figures S1, S2 and S3. Because protein sequences and chemical structures in the dark chemical genomics space could be significantly different from those for the known CPIs, predicting CPIs in the dark space is an archetypal, unaddressed OOD problem.

### 3.3 Portal Learning significantly outperforms state-of-the-art approaches to predicting dark CPIs

**Figure 4:**
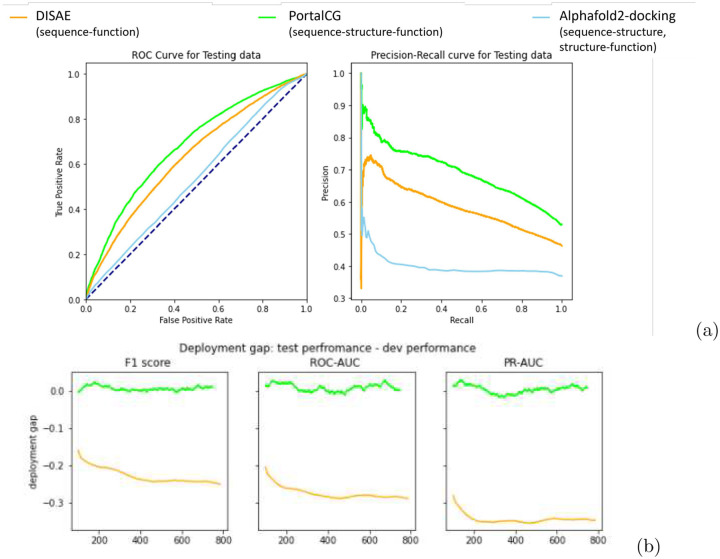
Comparison of PortalCG with the state-of-the-art method DISAE as baseline using the shifted evaluation test. (a) ROC and Precision-Recall curves for the “best” model instance selected by stress test; (b) Deployment gaps where the deployment gap of PortalCG is steadily around zero as training step increases while the deployment performance of DISAE deteriorates.

When compared with the state-of-the-art method DISAE[1], which already was shown to outperform other leading methods for predicting CPIs of orphan receptors, PortalCG demonstrates superior performance in terms of both Receiver Operating Characteristic (ROC) and Precision-Recall (PR) curves, as shown in Figure 4(a). Because the ratio of positive and negative cases is imbalanced, the PR curve is more informative than the ROC curve. The PR-AUC of PortalCG and DISAE is 0.714 and 0.603, respectively. In this regard, the performance gain of Portal Learning (18.4%) is significant (p-value < 1*e* – 40). Performance breakdowns for binding and non-binding classes can be found in Supplemental Figure S4.

PortalCG exhibits much higher recall and precision scores for positive cases (i.e., a chemical-protein pair that is predicted to bind) versus negative, as shown in Supplemental Figure S4; this is a highly encouraging result, given that there are many more negative (non-binding) than positive cases. The deployment gap, shown in Figure 4(b), is steadily around zero for PortalCG; this promising finding means that we can expect that, when applied to the dark genomics space, the performance will be similar to that measured using the development data set.

With the advent of high-accuracy protein structural models, predicted by AlphaFold2 [5], it now becomes feasible to use reversed protein-ligand docking (RPLD)[22] to predict ligand-binding sites and poses on dark proteins, on a genome-wide scale. In order to compare our method with the RPLD approach, blind docking to putative targets was performed via Autodock Vina[23]. After removing proteins that failed in the RPLD experiments (mainly due to extended structural loops), docking scores for 28,909 chemical-protein pairs were obtained. The performance of RPLD was compared with that of PortalGC and DISAE. As shown in Figure 4(a), both ROC and PR for RPLD are significantly worse than for PortalGC and DISAE. It is well known that PLD suffers from a high false-positive rate due to poor modeling of protein dynamics, solvation effects, crystallized waters, and other challenges [24]; often, small-molecule ligands will indiscriminately ‘stick’ to concave, pocket-like patches on protein surfaces. For these reasons, although AlphaFold2 can accurately predict many protein structures, the relatively low reliability of PLD still poses a significant limitation, even with a limitless supply of predicted structures [25]. Thus, the direct application of RPLD remains a challenge for predicting ligand binding to dark proteins. PortalCG’s end-to-end sequence-structure-function learning could be a more effective strategy: protein structure information is not used as a fixed input, but rather as an intermediate layer that can be tuned using various structural and functional information. From this perspective, again the role of protein structure in PortalCG can be seen as that of a portal (sequence→function; Figure 1) and a regularizer (Figure 2).

### 3.4 Both the STL and OOC-ML stages contribute to the improved performance of PortalCG

To gauge the potential contribution of each component of PortalCG to the overall system effectiveness in predicting dark CPIs, we systematically compared the four models shown in Table 2. Details of the exact model configurations for these experiments can be found in the Supplemental Materials Table S10 and Figure S13. As shown in Table 2, Variant 1, with a higher PR-AUC compared to the DISAE baseline, is the direct gain from transfer learning through 3D binding site information, all else being equal; yet, with transfer learning alone and without OOC-ML as an optimization algorithm in the target universe (i.e., Variant 2 versus Variant 1), the PR-AUC gain is minor. Variant 2 yields a 15% improvement while Variant 1 achieves only a 4% improvement. PortalCG (i.e., full Portal Learning), in comparison, has the best PR-AUC score. With all other factors held constant, the advantage of PortalCG appears to be the synergistic effect of both STL and OOC-ML. The performance gain measured by PR-AUC under a shifted evaluation setting is significant (p-value < 1e–40), as shown in Supplemental Figure S5.

We find that stress model selection is able to mitigate potential overfitting problems, as expected. Training curves for the stress model selection are in Supplemental Figures S4 and S6. As shown in Supplemental Figure S6, the baseline DISAE approach tends to over-fit with training, and IID-dev performances are all higher than PortalCG but deteriorate in OOD-test performance. Hence, the deployment gap for the baseline is −0.275 and −0.345 on ROC-AUC and PR-AUC, respectively, while PortalCG deployment is around 0.01 and 0.005, respectively.

### 3.5 Application of PortalCG to explore dark chemical genomics space

A production-level model using PortalCG was trained with ensemble methods for the deployment. Details are in the Supplemental Methods section. The trained PortalCG model was applied to two case-studies in order to assess its utility in the exploration of dark space. As long as a protein and chemical pair was presented to this model with their respective sequence and SMILES string, a prediction could be made, along with a corresponding prediction score. To select high confidence predictions, a histogram of prediction scores was built based on known pairs (Supplemental Figure S7). A threshold of 0.67, corresponding to a false positive rate of 2.18e-05, was identified to filter out high-confidence positive predictions. Around 6,000 drugs from the Drug Repurposing Hub[26] were used in the screening. The remainder of this section describes the two case-studies that were examined with PortalCG, namely (i) COVID-19 polypharmacology and (ii) the ‘undruggable’ portion of the human genome.

#### 3.5.1 COVID-19 polypharmacology

In order to identify lead compounds that may disrupt SARS-CoV-2-Human interactions, we screened 5,886 approved and investigational drugs against the 332 human proteins known to interact with SARS-CoV-2. We considered a drug-protein pair as a positive hit and selected it for further analysis only when all models in an ensemble vote as positive and the false positive rate does not exceed 2.18e-05. Drugs involved in these positive pairs were ranked according to the number of proteins to which they are predicted to bind. Detailed information is given in Supplemental Table S1. Most of these drugs are protein kinase inhibitors and are already in Phase 2 clinical trials. Among them, Fenebrutinib and NMS-P715 are predicted to bind to seven human SARS-CoV-2 interactors, as shown in Table 3. In order to elucidate how these drug molecules might associate with a SARS-CoV-2 interactor partner, we performed molecular docking for Fenebrutinib and NMS-P715. Structures of two SARS-CoV-2 interactors were obtained from the Protein Data Bank; the remaining five proteins do not have experimentally solved structures so their predicted structures (via AlphaFold2) were used for docking. For most of these structures, the binding pockets are unknown. Therefore, blind docking was employed, using Autodock Vina[23] to search the full surfaces (the accessible molecular envelope) and identify putative binding sites of Fenebrutinib and NMS-P715 on these interactors. Docking conformations with the best (lowest) predicted binding energies were selected for each protein; the respective binding energies are listed in Table 3.

**Figure 5:**
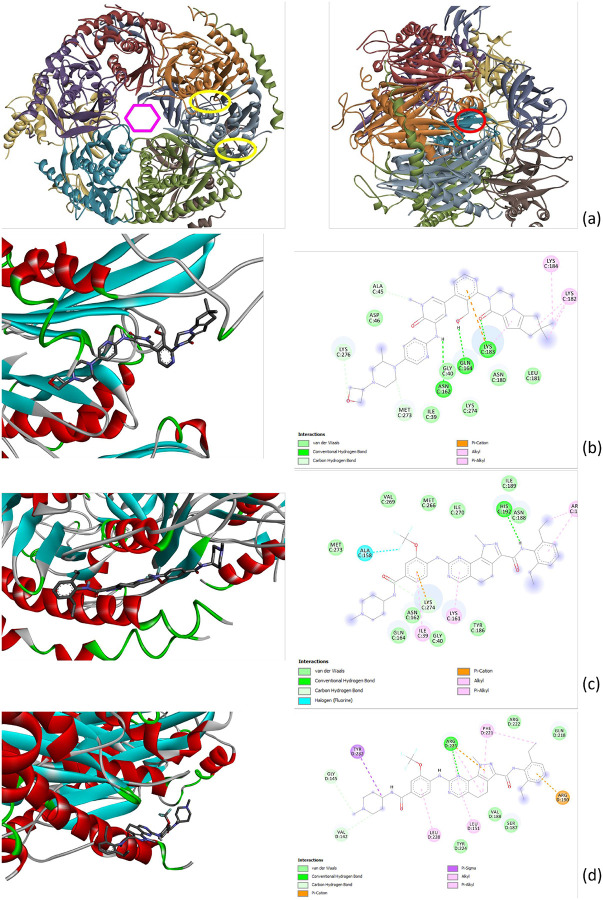
The 3D structure of the exosome complex and the binding conformations of Fenebrutinib and NMS-P715 on the complex components predicted by using Autodock: (a) The exosome complex structure; Left: yellow circles shows the binding pocket of NMS-P715 on RRP43 and RRP46, purple hexagon shows the gate; Right: red circle shows the binding pocket of Fenebrutinib on RRP43. (b) Fenebrutinib on RRP43. (c) NMS-P715 on RRP43. (d) NMS-P715 on RRP46.

Components of the exosome complex are predicted targets for both Fenebrutinib and NMS-P715. The exosome complex is a multi-protein, intracellular complex which is involved in degradation of many types of RNA molecules (e.g., via 3’→5’ exonuclease activities). As shown in Figure 5, the subunits of the exosomal assembly form a central channel; RNA passes through this region as part of the degradation/processing. Intriguingly, SARS-CoV-2’s genomic RNA has been found to be localized in the exosomal cargo, suggesting a key mechanistic role for the channel region in SARS-CoV-2 virion infectivity pathways [27]. Fenebrutinib and NMS-P715 were also predicted to bind to a specific exonuclease, RRP43, of the exosome complex, while NMS-P715 was also predicted to bind yet another exonuclease, RRP46.

The predicted binding poses for Fenebrutinib and NMS-P715 with the exosomal complex components are shown in Figure 5. The physicochemical/interatomic interactions between these two drugs and the exosome complex components are also schematized as a 2D layout in this figure. The favorable hydrogen bond, pi-alkyl, pi-cation and Van der Waals interactions provide additional support that Fenebrutinib and NMS-P715 do indeed bind to these components of the exosome complex. The predicted binding poses and 2D interactions maps for Fenebrutinib and NMS-P715 with other targeted proteins are shown in Supplementary Figures S8, S9, and S10.

#### 3.5.2 Illuminating the undruggable human genome

**Figure 6:**
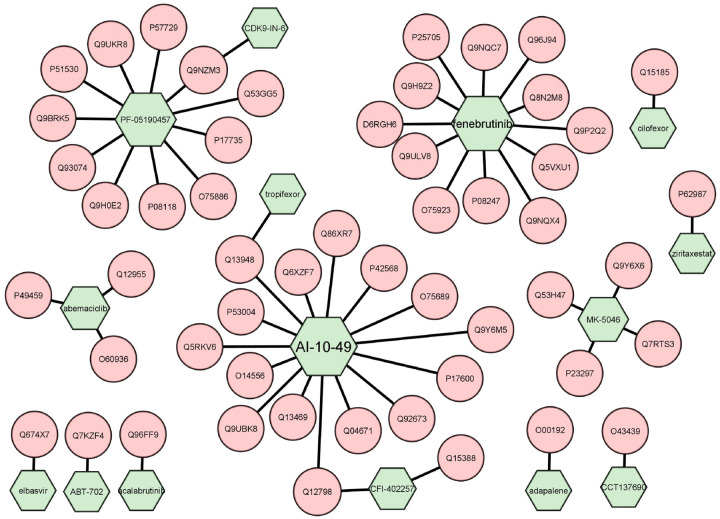
Drug-target interaction network for proteins associated with Alzheimer’s disease. Green represents drugs and pink represents targets.

It is well known that only a small subset of the human genome is considered druggable [28]. Many proteins are deemed “undruggable” because there is no information on their ligand-binding properties or other interactions with small-molecule compounds (be they endogenous or exogenous ligands). Here, we built an “undruggable” human disease protein database by removing the druggable proteins in Pharos [29] and Casas’s druggable proteins [30] from human disease associated genes [14] and applied PortalCG to predict the probability for these “undruggable” proteins to bind to drug-like molecules. A total of 12,475 proteins were included in our disease-associated undruggable human protein list. These proteins were ranked according to their probability scores, and 267 of them have a false positive rate lower than 2.18e-05, as listed in the supplementary material Table S2. Table 4 shows the statistically significantly enriched functions of these top ranked proteins as determined by DAVID [31]. The most enriched proteins are involved in alternative splicing of mRNA transcripts. Malfunctions in alternative splicing are linked to many diseases, including several cancers [32][33] and Alzheimer’s disease [34]. However, pharmaceutical modulation of alternative splicing process is a challenging task. Identifying new drug targets and their lead compounds for targeting alternative splicing pathways may open new doors to developing novel therapeutics for complex diseases with few treatment options. Diseases associated with these 267 human proteins were also listed in Table 5. Since one protein is always related to multiple diseases, these diseases are ranked by the number of their associated proteins. Most of top ranked diseases are related with cancer development. 21 drugs that are approved or in clinical development are predicted to interact with these proteins as shown in Table S3. Several of these drugs are highly promiscuous. For example, AI-10–49, a molecule that disrupts protein-protein interaction between CBFb-SMMHC and tumor suppressor RUNX1, may bind to more than 60 other proteins. The off-target binding profile of these proteins may provide invaluable information on potential side effects and opportunities for drug repurposing and polypharmacology. The drug-target interaction network built for predicted positive proteins associated with Alzheimer’s disease was shown in Figure 6. Functional enrichment, disease associations, and top ranked drugs for the undruggable proteins with well-studied biology (classified as Tbio in Pharos) and those excluding Tbio are list in Supplemental Table S4–S9.

## 4 Conclusion

This paper confronts the challenge of exploring dark chemical genomics space by recognizing it as an OOD generalization problem in machine learning, and by developing a new learning framework to treat this type of problem. We propose Portal Learning as a general framework that enables systematic control of the OOD generalization risk. As a concrete algorithmic example and use-case, PortalCG was implemented under the Portal Learning framework. Systematic examination of the PortalCG method revealed its superior performance compared to (i) a state-of-the-art deep learning model (DISAE), and (ii) an AlphaFold2-enabled, structure-based reverse docking approach. PortalCG showed significant improvements in terms of both sensitivity and specificity, as well as close to zero deployment performance gap. With this approach, we were able to explore the dark regions of the druggable genome. Applications of PortalCG to COVID-19 polypharmacology and to the targeting of hitherto undruggable human proteins affords novel new directions in drug discovery.

## 5 Methods

### 5.1 Full algorithm details

Portal learning as a system level framework involves collaborative new design from data preprocessing, data splitting to model architecture, model initialization, and model optimization and evaluation. The main illustrations are Figure 1 and Figure 2. Extensive explanation of each of the component and their motivations are available in Supplemental Materials section Method with Figure S11, and Algorithm1.

### 5.2 Data

PortalCG uses three database, Pfam[20], Protein Data Bank (PDB)[35] and ChEMBL[21]. Two applications are demonstrated, COVID-19 polypharmacology and undruggable human proteins, for which known approved drugs are collected from CLUE[26], 332 human proteins interacting SARS-CoV-2 are listed in recent publication[36], 12,475 undruggable proteins are collected by removing the druggable proteins in Pharos [29] and Casas’s druggable proteins [30] from human disease associated genes [14]. Detailed explanation of how each data set is used can be found in Supplemental Materials section 2.1.

Major data statistics are demonstrated in Figure 3 and Supplemental Materials Figure S1, S2, and S3.

### 5.3 Experiment implementation

Experiments are first organized to test PortalCG performance against baseline models, DISAE[1] and AlphFold2[5]. DISAE is a protein language which predicts protein function based on protein sequence information alone. AlphaFold2 uses protein sequence information to predict protein structure, combing docking methods, can be used to predict protein function. Main results are shown with Table 2 and Figure 4. Ablation studies is also performed mainly to test some variants of PortalCG components such as binding site distance prediction as shown in Supplemental Figure S12. Since Portal Learning is a general framework, there could be many interesting variants to pursue in future studies. To enhance application accuracy, a production level model is built with ensemble learning, and high confidence predictions are selected as demonstrated in Supplemental Material Figure S7. Evaluation metrics used are F1, ROC-AUC and PR-AUC.

Extensive details can be found in Supplemental Materials section 2.3, 2.4 and 2.5.

### 5.4 Related works

A literature review of related works could be found in Supplemental Materials section Related Works.

## Supplementary Material

Supplement 1

## Figures and Tables

**Table 1:** Data split for stress model instance selection

data split	Common practice	classic scheme applied in OOD	Portal learning	specification
train	IID train	IID train	/	each batch is from the same distribution
	/	/	OOD train	differentiate sub-distributions in each batch
dev	IID-dev	IID-dev	/	from the same distribution as the train set
	/	/	OOD-dev	from a different distribution from the training set
test	IID-test	/	/	from the same distribution as the training set
	/	OOD-test	OOD-test	from a different distribution from both OOD-dev and training set

**Table 2:** Ablation study of PortalCG.

	models	PR-AUC (OOD-test set)	ROC-AUC (OOD-test set)	PR-AUC Deployment gap	ROC-AUC Deployment gap
PortalCG	Portal learning	0.714±0.010	0.677±0.010	0.005±0.010	0.010±0.009
DISAE	PotalCG w/o STL or OOC-ML	0.603±0.005	0.636±0.004	−0.345±0.012	−0.275±0.016
variant 1	PotalCG w/o OOC-ML	0.629±0.005	0.661±0.004	/	/
variant 2	PotalCG w/o STL	0.698±0.015	0.654±0.062	/	/
Alphfold2-docking	/	0.398	0.535	/	/

**Table 3:** Docking scores for Fenebrutinib and NMS-P715

Docking scores of Fenebrutinib binding to predicted targets			
Uniprot ID	Protein name	PDB ID	Docking score (kcal/mol)
Q96B26	Exosome complex component RRP43	2NN6_C	−7.9
Q5JRX3	Presequence protease, mitochondrial	4L3T_A	−10.8
Q99720	Sigma non-opioid intracellular receptor 1	5HK1_A	−9.6
Q5VT66	Mitochondrial amidoxime-reducing component 1	6FW2_A	−10.4
P29122	Proprotein convertase subtilisin/kexin type 6	AF-P29122-F1 (157–622)	−8.5
Q96K12	Fatty acyl-CoA reductase 2	AF-Q96K12-F1 (1–478)	−10.1
O94973	AP-2 complex subunit alpha-2	AF-O94973-F1 (3–622)	−8.6
Docking scores of NMS-P715 binding to predicted targets			
Uniprot ID	Protein name	PDB ID	Docking score (kcal/mol)
Q9UN86	Ras GTPase-activating protein-binding protein 2	5DRV_A	−9.5
P67870	Casein kinase II subunit beta	1QF8_A	−8.6
Q96B26	Exosome complex component RRP43	2NN6_C	−9.3
P62877	E3 ubiquitin-protein ligase RBX1	2HYE_D	−7.9
P61962	DDB1- and CUL4-associated factor 7	AF-P61962-F1 (9–341)	−8.7
Q9NXH9	tRNA (guanine(26)-N(2))-dimethyltransferase	AF-Q9NXH9-F1 (53–556)	−9.0
Q9NQT4	Exosome complex component RRP46	2NN6_D	−8.6

**Table 4:** Functional Annotation enrichment for undruggable human disease proteins selected by PortalCG

David Functional Annotation enrichment analysis				
Enriched terms in UniProtKB keywords	Number of proteins involved	Percentage of proteins involved	P-value	Modified Benjamini p-value
Alternative splicing	171	66.5	7.70E-07	2.00E-04
Phosphoprotein	140	54.5	2.60E-06	3.40E-04
Cytoplasm	91	35.4	1.30E-05	1.10E-03
Nucleus	93	36.2	1.20E-04	8.10E-03
Metal-binding	68	26.5	4.20E-04	2.20E-02
Zinc	48	18.7	6.60E-04	2.90E-02

**Table 5:** Top ranked diseases associated with the undruggable human disease proteins selected by PortalCG

DiseaseName	# of undruggable proteins associated with disease
Breast Carcinoma	90
Tumor Cell Invasion	86
Carcinogenesis	83
Neoplasm Metastasis	75
Colorectal Carcinoma	73
Liver carcinoma	66
Malignant neoplasm of lung	56
Non-Small Cell Lung Carcinoma	56
Carcinoma of lung	54
Alzheimer’s Disease	54

## Data Availability

Data, a pre-trained PortalCG model, and PortalCG codes can be found in the following link: https://github.com/XieResearchGroup/PortalLearning
