# Supplementary material for: Exploration of Dark Chemical Genomics Space via Portal Learning: Applied to Targeting the Undruggable Genome and COVID-19 Anti-Infective Polypharmacology
Source: Res Sq. 2021 Dec 1:rs.3.rs-1109318. Preprint. [Version 1] doi: 10.21203/rs.3.rs-1109318/v1 (PMC8647653; doi:10.21203/rs.3.rs-1109318/v1)
Supplement: Supplement 1 [file 92f63dac604ba2136e10cff2.pdf]

# - **Supplemental Materials** -

Exploration of Dark Chemical Genomics Space via Portal Learning: Applied to Targeting the Undruggable Genome and COVID-19 Anti-infectious Polypharmacology

Tian Cai[1], Li Xie[2], Muge Chen[3], Yang Liu[2], Di He[1], Shuo Zhang[1], Cameron Mura[4], Philip E. Bourne[4], and Lei Xie[1,2,5,*]

[1] *Ph.D. Program in Computer Science, The Graduate Center, The City University of New York, New York, 10016, USA*
[2] *Department of Computer Science, Hunter College, The City University of New York, New York, 10065, USA*
[3] *Master Program in Computer Science, Courant Institute of Mathematical Sciences, New York University*
[4] *School of Data Science & Department of Biomedical Engineering, University of Virginia, Virginia, 22903, USA*
[5] *Helen and Robert Appel Alzheimer's Disease Research Institute, Feil Family Brain & Mind Research Institute, Weill Cornell Medicine, Cornell University, New York, 10021, USA*
[*] *lei.xie@hunter.cuny.edu*

November 24, 2021

Contents

1 Key terms **3**

2 Methods: PortalCG in four-universe configuration for dark chemical genomics space **5**

2.1 Data preprocessing . . . 5
2.2 Algorithm . . . 5
2.2.1 Chemical representation . . . 5
2.2.2 Protein sequence pre-training . . . 5
2.2.3 Protein structure regularization . . . 5
2.2.4 Out-of-cluster Meta Learning (OOC-ML) in protein function universe . . . 6
2.2.5 Stress model instance selection . . . 7
2.3 Implementation details . . . 7
2.4 Evaluation metrics . . . 7
2.5 Docking as baseline . . . 7
2.6 Production level for deployment . . . 8
2.7 Dark space exploration from a theoretical lens . . . 8

3 Additional tables **9**

Table S 1: Drugs predicted to interact with SARS-COVID-2 interactors . . . 9
Table S 2: Undruggable human disease-associated proteins selected by ProtalCG . . . 9
Table S 3: Chemicals interacted with undruggable human proteins . . . 14
Table S 4: Functional Annotation enrichment for human proteins in Tbio selected by PortalCG . . . 15
Table S 5: Top ranked diseases associated with proteins in Tbio selected by portal learning 16
Table S 6: Chemicals interacted with human proteins in Tbio . . . 16
Table S 7: Functional Annotation enrichment for undruggable human disease proteins without Tbio selected by PortalCG . . . 16
Table S 8: Top ranked diseases associated with undruggable human proteins excluding Tbio selected by portal learning . . . 16
Table S 9: Chemicals interacted with undruggable human proteins excluding Tbio . . . 16
Table S 10: Model architecture configuration . . . 16

4 Related works **19**

4.1 OOD generalization in deep learning . . . 19
4.2 Portal learning key components related . . . 19

5 Additional figures **21**

Figure S 1: Dark space statistics histogram . . . 21
Figure S 2: Dark space statistics trend line . . . 21
Figure S 3: Dark space statistics heatmap . . . 21
Figure S 4: Model performance breakdown to each class . . . 21
Figure S 5: Model performance t-test . . . 21
Figure S 6: Stress model selection performance curves against DISAE as baseline . . . 21
Figure S 7: Model performance histogram . . . 21
Figure S 8: The binding conformations and 2D-interactions of Fenebrutinib on the targeted proteins predicted by using Autodock . . . 21
Figure S 9: The binding conformations and 2D-interactions of Fenebrutinib on the targeted proteins predicted by using Autodock . . . 21
Figure S 10: The binding conformations and 2D-interactions of NMS-P715 on the targeted proteins predicted by using Autodock . . . 21
Figure S 11:Histogram equalization results . . . 21
Figure S 12: Ablation study . . . 21
Figure S 13: Model architecture illustration . . . 21

Reference **30**

1 Key terms

To provide common ground for discussion with readers of various backgrounds, we list the specification of key terms related to the methods in Supplemental materials. The following list provides explanations at an intuition level without the attempt to establish formal definitions. Readers could refer to referenced materials for more formal definitions.

Deep learning specific

1. model architecture: the design of a model as a set of trainable parameters without specification on the exact weight of the parameters [1].
2. loss landscape: the geometry of the global loss associated with a model architecture[2].
3. model instance: given a model architecture with certain amount of trainable parameters, a set of weights associated to the trainable parameters defines an instance of model; during the training process, each optimization step leads to a model instance.
4. optimization[1]: neural network is trained by optimizing a object function, usually in the form of minimizing a loss function.
5. global/local optimum: under the optimization formalization, the optimum point is at the end of the optimization process. As explained in [3], in an ideal world where the complete distribution of data is available to train a model, the optimum is global; while any stop point for any certain subdistribution is a local optimum.
6. model initialization: optimization always starts with an initialization of an model instance.
7. pretraining[4]: train a model on a pretext task before training on the target task; the trained model instances will become the initialization of the target task.
8. finetuning[4]: train a model on a target task with initialization pretrained task.
9. Independent and Identifically distributed (IID)[5]: if given a set of data x_i, each of these x_i observations is an independent draw from a fixed ("stationary") probability distribution.
10. Out-of-distribution (OOD) generalization[6]: Generalization consists in reducing the gap in performance between training data and testing data. When the data generating process from the training data is indistinguishable from that of the test data, it's "in-distribution", if not, it's called out-of-distribution generalization problem [6]. As in [7], considering datasets $Data_e := \{(x_i^e, y_i^e)\}_{i=1}^{n_e}$ collected under multiple domains e, each containing samples IID with a probability distribution $D(X^e, Y^e)$. The goal of OOD generalization is to use these datasets to learn a predictior $Y \approx f(X)$, which performs well across a large set of unseen but related domains $e \in \mathcal{E}_{all}$. Namely, the goal is to minimize

$$R^{OOD}(f) = \max_{e \in \mathcal{E}_{all}} R^e(f) \tag{1}$$

, where

$$R^e(f) := E_{X^e, Y^e}[l(f(X^e), Y^e)] \tag{2}$$

is the risk under domain e. Here the set $\mathcal{E}_{all}$ contains all possible domains.

11. generalization[1]: the most general goal of generalization is to enable the model make reliable prediction on unseen data; out-of-distribution prediction (OOD) is a more challenging type of generalization problem which requires the model to be generalizable to unseen data distribution.
12. mini-batch[1]: as a common practice for robustness and computation memory concerns, no matter how large a data set is available, only a sub set of data is sampled to train a model at each optimization step.
13. representation[1]: as coined by the line of work named "representation learning", the word representation is interachangable with "embedding", referring to a vector/matrix of learned features.
14. transfer model parameter: a technique that is related to pretraining-finuetuning; for the implementation, a simple initialization of part of the target model using the pretrained model could serve the purpose.

Chemical-protein interaction (CPI) specific

1. CPI prediction: formulated as a binary classification task, to predict whether or not a pair of protein and chemical will bind given only protein sequence and chemical SMILES string.
2. protein descriptor, chemical descriptor: the modules of a whole CPI prediction model to extract protein/chemical embedding in a euclidean space.

Portal learning specific

1. universe: a model architecture that defines a data transformation space, together with a data set.
2. portal: a model instance in a universe—which could be a local optimum in the current universe, but which facilitates moving the model to the global optimum in the ultimately targeted universe.
3. local loss landscape: optimize a model on a sub-distribution of the complete underlying distribution of the whole data set.
4. global loss landscape: the direction of gradient search towards global optimum for all sub-distributions.
5. stress test: a technique[8] to evaluate a predictor by observing its outputs on specifically designed inputs; three common types are stratified performance evaluation, shifted evaluation and contrastive evaluation.
6. shifted evaluation[8]: the stress test employed in this paper, which splits train/test data set by Pfam families, i.e. proteins in the testing and training sets come from different Pfam families. This is a simple simulation off dark space model deployment.
7. deployment gap: the difference between the performance evaluated by the test set and that evaluated by the development set.
8. classic deep learning training scheme: randomly split a whole data set into train/dev/test set; optimize model on a randomly sampled mini-batch data; choose a final trained model instance based on the best test evaluation metrics; usually adopts empirical risk minimization [9] formulation.

2 Methods: PortalCG in four-universe configuration for dark chemical genomics space

In this section, we present the detailed methodology used in portal learning in the context of a four-universe configuration.

2.1 Data preprocessing

The four-universe configuration is built on three major databases, Pfam[10], Protein Data Bank (PDB)[11], BioLp[12] and ChEMBL[13]. The data were preprocessed as follows.

- Protein sequence universe. All sequences from Pfam-A families are used to pretrain the protein descriptor following the same setting in DISAE [14] that highlights a MSA-based distillation process.
- Protein structure universe. In our protein structure data set, there are 30,593 protein structures, 13,104 ligands, and 91,780 ligand binding sites. Binding sites were selected according to the annotation from BioLip (updated to the end of 2020). Binding sites which contact with DNA/RNA and metal ions were not included. If a protein has more than one ligand, multiple binding pockets were defined for this protein. For each binding pocket, the distances between $C\alpha$ atoms of amino acid residues of the binding pocket were calculated. In order to obtain the distances between the ligand and its surrounding binding site residues, the distances between atom i in the ligand and each atom in the residue j of the binding pocket were calculated and the smallest distance wa selected as the distance between atom i and residue j. In order to get the sequence feature of the binding site residues in the DISAE protein sequence representation[14], binding site residues obtained from PDB structures (queries) were mapped onto the multiple sequence alignments of its corresponding Pfam family. First, a profile HMM database was built for the whole Pfam families. hmmscan [15] was applied to search the query sequence against this profile database to decide which Pfam family it belongs to. For those proteins with multiple domains, more than one Pfam families were identified. Then the query sequence was aligned to the most similar sequence in the corresponding Pfam family by using phmmer. Aligned residues on the query sequence were mapped to the multiple sequence alignments of this family according to the alignment between the query sequence and the most similar sequence.
- Chemical universe. All chemicals in the ChEMBL26 database consists of the chemical universe.
- Protein function universe. CPI classification prediction data is the whole ChEMBL26[13] database where the same threshold for defining positive and negative labels creating as that in DISAE [14] was used.

2.2 Algorithm

In the four-universe configuration, portal learning starts with portal identification in the protein sequence universe, then travels into protein structure universe for portal calibration before finally comes into the target protein function universe, where OOC-ML will be invoked for model optimization. Along the way, shifted evaluation, one type of stress model selection, is used to select the "best" model instance, which splits train/test based on Pfam families, i.e. training set and testing set have proteins come from different Pfam families. Each phase will be specified in the following sections.

2.2.1 Chemical representation

A chemical was represented as a graph and its embedding was learned using GIN[16].

2.2.2 Protein sequence pre-training

Protein descriptor is pretrained from scratch following exactly DISAE [14] on whole Pfam families, making it a universal protein language model. With standard Adam optimization, shifted evaluation is used to select the "best" instance.

2.2.3 Protein structure regularization

With the protein descriptor pretrained using the sequences from the whole Pfam, chemical descriptors and a distance learner were plugged in to fine-tune the protein representation. The distance learner follows Alphafold[17] which formulates a multi-way classification on a distrogram. Based on the

histogram of binding site distances, a histogram equalization[1] was applied to formulate a 10-way classification on our binding site structure data as in Supplemental material Figure S 11. Since protein and chemical descriptors output position-specific embeddings of a distilled protein sequence and all atoms of a chemical, pair-wise interaction features on the binding sites were created with a simple vector operation: a matrix multiplication was used to select embedding vectors of each binding residue and atom; multiply and broadcast the selected embedding vectors into a symmetric tensor as shown in the following, where H is embedding matrix of size $(number\_of\_residues, embedding_dimension)$ or $(number\_of\_atoms, embedding_dimension)$ and A is selector matrix[18],

$$H^{protein}_{binding\_site} = A^{protein} * H^{protein}_{full\_distilled}$$

$$H^{chemical}_{binding\_site} = A^{chemical} * H^{chemical}_{full\_chemical\_graph}$$

$$H^{interaction}_{binding\_site} = (H^{protein}_{binding\_site})^T * H^{chemical}_{binding\_site}$$

This pair-wise interaction feature tensor $H^{interaction}_{binding\_site}$ was fed into a Attentive Pooling[19] layer followed by feed-forward layer for final 10-way classification. Detailed model architecture configuration could be found in Table S 10 and Figure S13 .The intuition for the simplest form of distance learner is to put all stress of learning on the shared protein and chemical descriptors which will carry information across universes. Again, with standard Adam optimization, shifted evaluation was used to select the "best" instance. Two versions of distance structure prediction were implemented, one formulated as a binary classification, i.e. contact prediction, one formulated as a multi-way classification, i.e. distogram prediction. The performance of the two version are similar, as shown in Figure S 12.

2.2.4 Out-of-cluster Meta Learning (OOC-ML) in protein function universe

With fine-tuned protein descriptor in the protein function universe, a binary classifier is plugged on, which is a ResNet[20] layered with two linear layers as shown in Table S 10 and Figure S13. What plays the major role in this phase is the optimization algorithm OOC-ML as shown in pseudocode **Algorithm1** and main content Figure 1(B),(C.1). The local loss landscape exploration is reflected in line 4-9, and line 10 shows ensemble of global loss landscape. Note that more variants could be derived from changing sampling rule (line 3 and 5) and global loss ensemble rule.

OOC-ML is built on MAML[21] but has significant differences. Echoing to steps illustrated in the Figure 1 of the main text:

1. As shown in main content Figure1 (B), OOC-ML has a sub-distribution data split into support set and query set, or as MAML named it, meta-train and meta-test within training set and test set. However, MAML sub-distributions are identified from the label space $\{Y\}$ while OOC-ML identifies sub-distributions, i.e. clusters, from input feature space $\{X\}$. In PortalCG, the clusters are identified by Pfam. Further, OOC-ML allows the utilization of very small clusters where very limited known data points are available for training. For example, in PortalCG, some Pfam families with too few samples to be split into support and query set are organized as query-set-alone, which participate only in the global loss optimization, as detailed below.

2. In each mini-batch, a few sub-distributions are sampled. The whole optimization has two layers, inner loop and outer loop. At the inner loop, each sub-distribution data has its own local loss landscape. The support set is used for in-distribution optimization on the local loss landscape.

3. The locally optimized model is then used on query set to get a query set loss, which will be fed to the global loss landscape. Each sub-distribution is independently optimized. This step is the same as MAML. What is different is that OOC-ML also calculates query-set without local in-distribution optimization for the small clusters.

4. Local query set losses are pooled together and the model will be optimized on the global loss landscape as meta-optimization defined in MAML.

5. After finishing train, the model will be deployed.

6. MAML is designed for multi-class classification in few-shot learning, at deployment stage, it's expected to meet new unseen class. And it's assumed that there are **a few labelled sample available** as support set, hence named as **few-shot learning**. For each unseen class, the trained model will carry out a fast in-distribution adaptation using support set before final prediction on the query set. However, this is impossible in the context of dark space illumination. Portal learning trained model has to make robust predictions without any chance of in-distribution adaptation.

[1] Histogram equalization: https://en.wikipedia.org/wiki/Histogram_equalization

Algorithm 1: Portal Learning Optimization Algorithm: Out-of-cluster Meta-learning

input : $p(\mathbf{D})$, CPI data distribution over whole pfams, where each $D_i \in \mathbf{D}$ is a set of CPI pairs for the $pfam_i$;
α, β, learning step size hyperparameters;
L, number of optimization steps in each round of local exploration;
T, number of global training steps;
K, number of points sampled from a local neighborhood

output: θ the whole model weights

initialization whole model weights θ (with weight transfered from portal for protein and chemical descriptors and random initiliazed weights for binary classifier)
for t *in* T **do**
  Sample a $D_i \sim p(D)$;
  for l *in* L **do**
    Sample a positive-negative balanced mini-batch of K pairs $neighborhood_m \sim D_i$;
    for $point_j$ *in* $neighborhood_m$ **do**
      Evaluate $\nabla_\theta L_{point_j}(f_\theta)$ with respect to K examples;
      Compute adapted parameters with gradient decent:$\theta_i^{'} = \theta - \alpha \nabla_\theta L_{point_j}(f_\theta)$;
    end
    Update $\theta \leftarrow \theta - \beta \nabla_\theta \sum_{D_i \sim p(D)} L_{point_j}(f_\theta^{'})$;
  end
end

2.2.5 Stress model instance selection

In classic training scheme common practice, there are 3-split data sets, "train set", "dev set" and "test set". Train set as the name suggested is used to train model. Test set as commonly expected is used to set an expectation of performance when applying the trained model to unseen data. Dev set is to select the preferred model instance. In OOD setting, data is split (main content Table 1) such that dev set is a OOD from train set and test set is a OOD from both train and dev set. Deployment gap is calculated by deducting ODD-dev performance with OOD-test performance.

2.3 Implementation details

With portal learning being a framework, all experiments are based on the configuration of a four-universe design. Four major variants of models are trained as shown in main content Table 2 for controlled factor experiments to verify the contribution of key components of Portal Learning. In this section we present implementation details.

Due to the large number of total samples, all training are carried out under global step-based formalization instead of epoch-based. Typically, a deep learning model is trained for numerous epochs, in each epoch the model will loop over all training data. Evaluation will be carried out once on the whole test data set at the end of each epoch. In the global step formalization, a mini-batch is sampled at uniform random from pre-split training data set. For a pre-defined total number of global steps, this mini-batch sampling will be repeated. Training is stopped when loss decreases are within a pre-defined error margin. To evaluate along the way of training, for every m global steps of training, a subset of test data is sampled uniformly randomly from a pre-split test set. To compute generalization gaps, in addition to evaluate on test set split according to the shifted evaluation, a dev set is held out from the train set for the evaluation as well. In this way, dev set and train set are iid. The performance difference between dev and train is the *observed space generalization gap* while the performance difference between dev and test is the *dark space generalization gap.*

2.4 Evaluation metrics

Distogram prediction uses an average accuracy on the distogram. CPI binary classification uses F1, ROC-AUC and PR-AUC for overall evaluation with breakdown by class F1, recall and precision scores.

2.5 Docking as baseline

Protein-ligand docking was performed using Autodock Vina[22]. The whole protein surface search implemented in the Autodock Vina was applied to identify the ligand binding pocket. The center of each protein was set as the center of the binding pocket. The largest distance of the protein atoms to the center of the protein is calculated for each x, y, and z direction to define the edge of each

protein. 10 Angstrom of extra space was added to the protein edge to set up the search space for the docking.

2.6 Production level for deployment

To create a production level model, three models were trained in PortalCG with only difference in data split. Dev set was OOD in respect of training set to make sure there was no overlapped Pfam families between them. By rotating Pfam families between training set and OOD-dev set in the fashion of a cross-validation, each of the three models was trained on different train set in light of Pfam families involved. Then a voting mechanism was used to make the final prediction.

2.7 Dark space exploration from a theoretical lens

A neural network classifier is trained by minimizing a loss function with a standard form as the following:

$$L(\theta) = \frac{1}{|D_t|} \sum_{(x,y)\in D_t} -\log p_\theta(x,y)$$

where $p_\theta(x,y)$ is the probability that a sample x belongs to the class y according to the trained neural network with parameters θ, and D_t is the training data set with the number of samples $|D_t|$. As laid out in the recent framework in [3] that reasons about generalization in deep learning, the test error of a model f_t could be decomposed as follows,

$$TestError(f_t) = \underbrace{TestError(f_t^{iid})}_{\text{ideal world}} + \underbrace{[TestError(f_t) - TestError(f_t^{iid})]}_{\text{real world generalization gap}}$$

When data are sampled as independent and identically distributed (iid) random variables, “ideal world” is a scenario where the complete data distribution is available with infinite data and optimization is on a population loss landscape. By contrast, “real world” has only finite data, where optimization is on an empirical loss landscape. In the dark space context of the OOD setting, this decomposition needs to be changed to

$$TestError(f_t^{OOD}) = \underbrace{TestError(f_t^{iid})}_{\text{observed space}} + \underbrace{[TestError(f_t^{OOD}) - TestError(f_t^{iid})]}_{\text{dark space generalization gap}}$$

and

$$TestError(f_t^{iid}) = TrainError(f_t^{iid}) + \underbrace{[TestError(f_t)^{iid} - TrainError(f_t^{iid})]}_{\text{observed space generalization gap}}$$

This explains that the effort could be devoted to decrease the observed space error and/or the dark space generalization gap to reduce $TestError(f_t^{OOD})$.

$$\nabla_\theta J(\theta) \approx g = \frac{1}{m}\sum_{i=1}^{m} \nabla_\theta L(x^{(i)}, y^{(i)}, \theta)$$

$$\theta \leftarrow \theta - \epsilon g$$

When stochastic gradient descent (SDG) is applied to the optimization, it approximately estimates $\nabla_\theta J(\theta)$, the expectation of gradient, using a small set of sample of size m, i.e., the mini-batch drawn uniformly from the training set. When all data are IID, this approximation works fine to update θ with g. However, for the ODD with unknown distribution, this θ updating function could easily fall into a local minimum based on the m mini-batch samples.

The test error for a trained model in the OOD setting includes two parts: test errors in the observed IID space and a generalized gap when stepping into the OOD space. Furthermore, as discussed and proved in [6], [7], not all OOD tasks are equal. Depending on how different the OOD data set is from the train set, some OOD task could be more challenge. It is true for predicting ligand binding to dark proteins. It is impossible for training data to provide sufficient coverage of the whole distribution in the dark chemical genomics space. The motivation of Portal Learning for exploring the dark space follows: one model architecture defines a functional mapping space, together with a data set defines a **universe**. The model initialized instance in a universe closer to the global optimum universe is a **portal** that is transferred from an associated universe. CPI dark space is impossible to be explored if the learning is confined only in the observed protein function, i.e. CPI universe since the known data are far sparse as shown in main content Figure 3. Hence STL is important to identify portals. The model optimization on a loss function can decrease IID training errors but will not help with the observed IID space generalization gap $TestError(f_t)^{iid} - TrainError(f_t^{iid})$ or the dark space generalization gap $TestError(f_t^{OOD}) - TestError(f_t^{iid})$. With Portal Learning, stress model instance selection can narrow the first gap and OOC-ML can narrow the second gap.

3 Additional tables

IhChIKey	Number of hits	Drug name	Clinical trail	Mechanism of Action
WNEODWDFDXWOLU-QHCPKHFHSA-N	7	fenebrutinib	phase 2	Bruton's tyrosine kinase (BTK) inhibitor
JFOAJUGFHDCBJJ-UHFFFAOYSA-N	7	NMS-P715	preclinical	protein kinase inhibitor
QHLVBNKYJGBCQJ-UHFFFAOYSA-N	4	NMS-1286937	phase 2	PLK inhibitor
FUXVKZWTXQUGMW-FQEVSTJZSA-N	4	9-aminocamptothecin	phase 2	topoisomerase inhibitor
DKZYXHCYPUVGAF-JCNLHEQBSA-N	2	OTS167	phase 1/phase 2	maternal embryonic leucine zipper kinase inhibitor
VYLOOGHLKSNNEK-PIIMJCKOSA-N	1	tropifexor	phase 2	FXR agonist
TZKBVRDEOITLRB-UHFFFAOYSA-N	1	GZD824	preclinical	Bcr-Abl kinase inhibitor
KZSKGLFYQAYZCO-UHFFFAOYSA-N	1	cilofexor	phase 3	FXR agonist

Table S 1: Drugs predicted to interact with SARS-COVID-2 interactors

Uniprot	Protein name	Drug name	Probscore
Q8TEX9	Importin-4	fenebrutinib	0.68089414
Q8NB66	Protein unc-13 homolog C	AI-10-49	0.67951804
Q9NZF1	Placenta-specific gene 8 protein	AI-10-49	0.6778485
Q96G03	Phosphoglucomutase-2	fenebrutinib	0.677423
Q8IWU4	Zinc transporter 8	fenebrutinib	0.677423
P53004	Biliverdin reductase A	AI-10-49	0.6769635
P40879	Chloride anion exchanger	fenebrutinib	0.67688245
Q8IYL2	Probable tRNA	fenebrutinib	0.6768823
Q9ULL4	Plexin-B3	AI-10-49	0.67652583
Q96SZ6	Mitochondrial tRNA methylthiotransferase CDK5RAP1	fenebrutinib	0.6763639
Q9BRT9	DNA replication complex GINS protein SLD5	fenebrutinib	0.6763639
Q8N2U0	Transmembrane protein 256	CCT137690	0.67634773
Q9UC06	Zinc finger protein 70	fenebrutinib	0.6761195
Q9BPX5	Actin-related protein 2/3 complex subunit 5-like protein	Q-203	0.67571765
Q8TDF6	RAS guanyl-releasing protein 4	NMS-1286937	0.6756182
Q9P2G3	Kelch-like protein 14	NMS-1286937	0.6756182
Q9HBT7	Zinc finger protein 287	CCT137690	0.6752016
Q9UKR8	Tetraspanin-16	PF-05190457	0.6751168
P59044	NACHT, LRR and PYD domains-containing protein 6	MK-5046	0.67498016
Q16774	Guanylate kinase	MK-5046	0.6749801
Q9HCE5	N6-adenosine-methyltransferase non-catalytic subunit	PF-05190457	0.67497396
Q15185	Prostaglandin E synthase 3	cilofexor	0.6745854
Q8WUA7	TBC1 domain family member 22A	fenebrutinib	0.67439413
Q17RS7	Flap endonuclease GEN homolog 1	CGM097	0.67434615
Q14244	Ensconsin	PF-05190457	0.67431164
Q9BRK5	45 kDa calcium-binding protein	PF-05190457	0.67431164
Q9UKJ5	Cysteine-rich hydrophobic domain-containing protein 2	CCT137690	0.67429835
Q6ZWJ1	Syntaxin-binding protein 4	AI-10-49	0.6742442
Q9HAR2	Adhesion G protein-coupled receptor L3	AI-10-49	0.67414063
P98161	Polycystin-1	AI-10-49	0.67414063
Q92673	Sortilin-related receptor	AI-10-49	0.6740716
D6RGH6	Multicilin	fenebrutinib	0.67397785
Q8NHH9	Atlastin-2	fenebrutinib	0.67395777
Q9H0E2	Toll-interacting protein	PF-05190457	0.6739072
O15145	Actin-related protein 2/3 complex subunit 3	PF-05190457	0.6739072
P51116	Fragile X mental retardation syndrome-related protein 2	abemaciclib	0.6738332
Q9BR09	Neuralized-like protein 2	elbasvir	0.67369795
P42568	Protein AF-9	AI-10-49	0.67364717
P17600	Synapsin-1	AI-10-49	0.67364717
P48553	Trafficking protein particle complex subunit 10	AI-10-49	0.6736086
Q12955	Ankyrin-3	abemaciclib	0.6735056

Table 2 continued from previous page

Uniprot	Protein name	Drug name	Probscore
O60936	Nucleolar protein 3	abemaciclib	0.6735056
Q02575	Helix-loop-helix protein 1	AI-10-49	0.6734969
P49640	Homeobox even-skipped homolog protein 1	CFI-402257	0.67348343
P22670	MHC class II regulatory factor RFX1	PF-05190457	0.67346805
Q8IUF8	Ribosomal oxygenase 2	NMS-1286937	0.67343307
P22681	E3 ubiquitin-protein ligase CBL	NMS-1286937	0.67343307
Q7RTS3	Pancreas transcription factor 1 subunit alpha	MK-5046	0.67334837
Q9Y5L4	Mitochondrial import inner membrane translocase subunit Tim13	NMS-P715	0.6733338
P17735	Tyrosine aminotransferase	PF-05190457	0.673238
O95294	RasGAP-activating-like protein 1	PF-05190457	0.673224
Q8NCD3	Holliday junction recognition protein	PF-05190457	0.673224
Q86W28	NACHT, LRR and PYD domains-containing protein 8	MK-5046	0.6731598
Q04671	P protein	AI-10-49	0.67310166
Q9C035	Tripartite motif-containing protein 5	AI-10-49	0.6731016
Q66K64	DDB1- and CUL4-associated factor 15	AI-10-49	0.672944
Q5T9A4	ATPase family AAA domain-containing protein 3B	CFI-402257	0.67286545
Q96MP8	BTB/POZ domain-containing protein KCTD7	CFI-402257	0.6728654
Q9UIE0	Zinc finger protein 230	CFI-402257	0.672853
Q3SYG4	Protein PTHB1	abemaciclib	0.67280704
Q8IUY3	GRAM domain-containing protein 2A	AI-10-49	0.67273754
O75689	Arf-GAP with dual PH domain-containing protein 1	AI-10-49	0.67273754
Q9NZP8	Complement C1r subcomponent-like protein	fenebrutinib	0.6727066
Q9NQX4	Unconventional myosin-Vc	fenebrutinib	0.6727064
Q15388	Mitochondrial import receptor subunit TOM20 homolog	CFI-402257	0.6726911
Q9NXL9	DNA helicase MCM9	piperaquine-phosphate	0.6726752
P53370	Nucleoside diphosphate-linked moiety X motif 6	piperaquine-phosphate	0.6726752
Q8N7C0	Leucine-rich repeat-containing protein 52	PF-05190457	0.67262363
O95231	Homeobox protein VENTX	NMS-P715	0.67261267
Q9HC56	Protocadherin-9	AI-10-49	0.6725752
A6NNW6	Enolase 4	AI-10-49	0.6725752
P35612	Beta-adducin	CFI-402257	0.67255336
Q9Y6M5	Zinc transporter 1	AI-10-49	0.67255074
Q01804	OTU domain-containing protein 4	AI-10-49	0.6725507
Q9Y678	Coatomer subunit gamma-1	MK-5046	0.67254347
Q9BTY7	Protein HGH1 homolog	AI-10-49	0.67243195
Q8NEM0	Microcephalin	CFI-402257	0.6724283
Q7LGA3	Heparan sulfate 2-O-sulfotransferase 1	AI-10-49	0.67242616
O75956	Cyclin-dependent kinase 2-associated protein 2	ziritaxestat	0.6723895
P62987	Ubiquitin-60S ribosomal protein L40	ziritaxestat	0.6723893
Q14257	Reticulocalbin-2	CDK9-IN-6	0.6723883
Q9H0I3	Coiled-coil domain-containing protein 113	tropifexor	0.6723477
P32019	Type II inositol 1,4,5-trisphosphate 5-phosphatase	fenebrutinib	0.6723469
O95409	Zinc finger protein ZIC 2	fenebrutinib	0.6723469
P56179	Homeobox protein DLX-6	fenebrutinib	0.67233485
Q12798	Centrin-1	AI-10-49	0.6722851
Q14714	Sarcospan	AI-10-49	0.6722851
Q96LI5	CCR4-NOT transcription complex subunit 6-like	abemaciclib	0.6722749

Table 2 continued from previous page

Uniprot	Protein name	Drug name	Probscore
Q86XR7	TIR domain-containing adapter molecule 2	AI-10-49	0.6722719
P53672	Beta-crystallin A2	CCT137690	0.6722543
O43439	Protein CBFA2T2	CCT137690	0.6722543
Q9H0E3	Histone deacetylase complex subunit SAP130	fenebrutinib	0.6722474
Q8NFZ0	F-box DNA helicase 1	fenebrutinib	0.6722474
Q8N0S2	Synaptonemal complex central element protein 1	CFI-402257	0.67223567
Q92667	A-kinase anchor protein 1, mitochondrial	fenebrutinib	0.67222863
O60884	DnaJ homolog subfamily A member 2	PF-05190457	0.6721347
P41214	Eukaryotic translation initiation factor 2D	AI-10-49	0.6721203
Q6R327	Rapamycin-insensitive companion of mTOR	AI-10-49	0.67211396
O75886	Signal transducing adapter molecule 2	PF-05190457	0.67206836
Q9UMX6	Guanylyl cyclase-activating protein 2	PF-05190457	0.6720683
Q99447	Ethanolamine-phosphate cytidylyltransferase	CCT137690	0.67205685
Q6FI13	Histone H2A type 2-A	PF-05190457	0.6720233
Q969S2	Endonuclease 8-like 2	fenebrutinib	0.67200994
Q6XZF7	Dynamin-binding protein	AI-10-49	0.67196065
Q96EX1	Small integral membrane protein 12	fenebrutinib	0.6719141
Q8TD57	Dynein axonemal heavy chain 3	fenebrutinib	0.6719141
O15375	Monocarboxylate transporter 6	PF-05190457	0.6719058
Q9H7E2	Tudor domain-containing protein 3	NMS-1286937	0.6718896
A6NI73	Leukocyte immunoglobulin-like receptor subfamily A member 5	AI-10-49	0.671879
Q9NZM3	Intersectin-2	CDK9-IN-6	0.6718554
O75145	Liprin-alpha-3	CDK9-IN-6	0.6718554
Q9NSD9	Phenylalanine–tRNA ligase beta subunit	abemaciclib	0.6718319
O15195	Villin-like protein	abemaciclib	0.6718319
P05538	HLA class II histocompatibility antigen, DQ beta 2 chain	cilofexor	0.671822
Q8TD57	Dynein axonemal heavy chain 3	cilofexor	0.671822
Q9NRD9	Dual oxidase 1	abemaciclib	0.6718094
Q8WUQ7	Cactin	AI-10-49	0.67178315
Q9H7C4	Syncoilin	AI-10-49	0.67178315
Q70EK9	Ubiquitin carboxyl-terminal hydrolase 51	PF-05190457	0.671742
O43837	Isocitrate dehydrogenase [NAD] subunit beta, mitochondrial	PF-05190457	0.67174196
Q9UKY1	Zinc fingers and homeoboxes protein 1	CFI-402257	0.6717407
Q6ZMJ2	Scavenger receptor class A member 5	CFI-402257	0.6717407
P61371	Insulin gene enhancer protein ISL-1	fenebrutinib	0.6717256
Q6NSZ9	Zinc finger and SCAN domain-containing protein 25	fenebrutinib	0.6717256
Q92754	Transcription factor AP-2 gamma	IACS-10759	0.67171496
Q5RKV6	Exosome complex component MTR3	AI-10-49	0.6716919
Q9BRA0	N-alpha-acetyltransferase 38, NatC auxiliary subunit	MK-5046	0.6716785
Q9BSF8	BTB/POZ domain-containing protein 10	CCT137690	0.67165
Q6PJ69	Tripartite motif-containing protein 65	AI-10-49	0.6716369
Q9NRP0	Oligosaccharyltransferase complex subunit OSTC	AI-10-49	0.6716369
Q8TBM7	Transmembrane protein 254	PF-05190457	0.6716311
Q674X7	Kazrin	elbasvir	0.6716015
Q9Y3C4	EKC/KEOPS complex subunit TPRKB	abemaciclib	0.6715913
P59190	Ras-related protein Rab-15	abemaciclib	0.6715913
Q9NZM3	Intersectin-2	PF-05190457	0.6715465
P05455	Lupus La protein	AI-10-49	0.6715072
Q96KN3	Homeobox protein PKNOX2	abemaciclib	0.67148525

Table 2 continued from previous page

Uniprot	Protein name	Drug name	Probscore
P54278	Mismatch repair endonuclease PMS2	abemaciclib	0.6714852
P49069	Guided entry of tail-anchored proteins factor CAMLG	fenebrutinib	0.6714454
Q8IYT4	Katanin p60 ATPase-containing subunit A-like 2	AI-10-49	0.6714413
Q8WVI0	Small integral membrane protein 4	AI-10-49	0.67144126
Q9Y6X6	Unconventional myosin-XVI	MK-5046	0.6713607
Q8N357	Solute carrier family 35 member F6	MK-5046	0.6713607
Q96BP2	Coiled-coil-helix-coiled-coil-helix domain-containing protein 1	abemaciclib	0.671357
Q9UJQ4	Sal-like protein 4	MK-5046	0.67135173
Q9HCQ5	Polypeptide N-acetylgalactosaminyltransferase 9	fenebrutinib	0.67132807
Q9P2Q2	FERM domain-containing protein 4A	fenebrutinib	0.67132807
Q7Z7M8	UDP-GlcNAc:betaGal beta-1,3-N-acetylglucosaminyltransferase 8	NMS-P715	0.6713157
Q9HCK5	Protein argonaute-4	PF-05190457	0.6713007
O95405	Zinc finger FYVE domain-containing protein 9	AI-10-49	0.67127687
Q92911	Sodium/iodide cotransporter	PF-05190457	0.67127484
Q8WWI1	LIM domain only protein 7	fenebrutinib	0.6712641
Q8N3C7	CAP-Gly domain-containing linker protein 4	fenebrutinib	0.671264
Q9UBS8	E3 ubiquitin-protein ligase RNF14	PF-05190457	0.671259
Q9BY66	Lysine-specific demethylase 5D	PF-05190457	0.671259
Q9UPM8	AP-4 complex subunit epsilon-1	abemaciclib	0.6712124
Q9BYE0	Transcription factor HES-7	MK-5046	0.6711568
Q9BSD7	Cancer-related nucleoside-triphosphatase	AI-10-49	0.67114973
Q9NXH9	tRNA	tropifexor	0.6711371
Q13948	Protein CASP	tropifexor	0.6711371
Q9C019	Tripartite motif-containing protein 15	abemaciclib	0.67111254
Q5TA50	Ceramide-1-phosphate transfer protein	ABT-702	0.6711099
Q7KZF4	Staphylococcal nuclease domain-containing protein 1	ABT-702	0.6711099
P08247	Synaptophysin	fenebrutinib	0.6711092
A4IF30	Solute carrier family 35 member F4	AI-10-49	0.67109805
Q9NRA8	Eukaryotic translation initiation factor 4E transporter	AI-10-49	0.67109805
Q96FF9	Sororin	acalabrutinib	0.6710895
P10073	Zinc finger and SCAN domain-containing protein 22	PF-05190457	0.67107534
P0CW19	LIM and senescent cell antigen-like-containing domain protein 3	PF-05190457	0.67107534
Q9UPM8	AP-4 complex subunit epsilon-1	fenebrutinib	0.6710706
P08118	Beta-microseminoprotein	PF-05190457	0.6710468
Q96P16	Regulation of nuclear pre-mRNA domain-containing protein 1A	fenebrutinib	0.6710315
P62280	40S ribosomal protein S11	piperaquine-phosphate	0.6710226
Q8TE49	OTU domain-containing protein 7A	AI-10-49	0.6710194
Q53HI1	Protein unc-50 homolog	AI-10-49	0.6710194
Q8WW32	High mobility group protein B4	PF-05190457	0.6709929
P57729	Ras-related protein Rab-38	PF-05190457	0.67099285
Q8N2M8	CLK4-associating serine/arginine rich protein	fenebrutinib	0.6709883
P25705	ATP synthase subunit alpha, mitochondrial	fenebrutinib	0.6709781
Q9Y5H3	Protocadherin gamma-A10	PF-05190457	0.6709645
Q9NU63	Zinc finger protein 57 homolog	fenebrutinib	0.67096215
P43363	Melanoma-associated antigen 10	AI-10-49	0.6709133

Table 2 continued from previous page

Uniprot	Protein name	Drug name	Probscore
Q13469	Nuclear factor of activated T-cells, cytoplasmic 2	AI-10-49	0.670904
Q8NG77	Olfactory receptor 2T12	AI-10-49	0.670904
Q9Y2G8	DnaJ homolog subfamily C member 16	PF-05190457	0.67090386
Q9ULV8	E3 ubiquitin-protein ligase CBL-C	fenebrutinib	0.67089945
Q96EF0	Myotubularin-related protein 8	elbasvir	0.67085487
Q8TD16	Protein bicaudal D homolog 2	abemaciclib	0.6708334
P49459	Ubiquitin-conjugating enzyme E2 A	abemaciclib	0.6708334
Q6XZF7	Dynamin-binding protein	AI-10-49	0.6708271
Q9Y4I1	Unconventional myosin-Va	AI-10-49	0.6708271
Q96FL9	Polypeptide N-acetylgalactosaminyltransferase 14	MK-5046	0.67080116
Q9HD67	Unconventional myosin-X	MK-5046	0.6708011
Q13214	Semaphorin-3B	MK-5046	0.6707926
Q53H47	Histone-lysine N-methyltransferase SETMAR	MK-5046	0.6707926
P46776	60S ribosomal protein L27a	AI-10-49	0.67078424
Q15306	Interferon regulatory factor 4	AI-10-49	0.6707842
Q2TAA2	Isoamyl acetate-hydrolyzing esterase 1 homolog	abemaciclib	0.67076474
O43324	Eukaryotic translation elongation factor 1 epsilon-1	PF-05190457	0.67074585
Q96R27	Olfactory receptor 2M4	fenebrutinib	0.6707435
Q5VXU1	Sodium/potassium-transporting ATPase subunit beta-1-interacting protein 2	fenebrutinib	0.6707435
Q5VWQ8	Disabled homolog 2-interacting protein	fenebrutinib	0.67072076
Q9UJF2	Ras GTPase-activating protein nGAP	elbasvir	0.67071134
O75923	Dysferlin	fenebrutinib	0.6707069
O75592	E3 ubiquitin-protein ligase MYCBP2	PF-05190457	0.6706958
Q14714	Sarcospan	CFI-402257	0.67069536
Q12798	Centrin-1	CFI-402257	0.67069536
Q9H9Z2	Protein lin-28 homolog A	fenebrutinib	0.67065567
Q8N2A8	Mitochondrial cardiolipin hydrolase	fenebrutinib	0.67065555
Q5VV41	Rho guanine nucleotide exchange factor 16	abemaciclib	0.6706521
Q96QT6	PHD finger protein 12	fenebrutinib	0.67064524
Q13948	Protein CASP	AI-10-49	0.6706354
Q9NXH9	tRNA	AI-10-49	0.6706354
Q969E2	Secretory carrier-associated membrane protein 4	fenebrutinib	0.67062545
P58107	Epiplakin	fenebrutinib	0.67062545
P55036	26S proteasome non-ATPase regulatory subunit 4	NMS-P715	0.67059726
Q11128	4-galactosyl-N-acetylglucosaminide 3-alpha-L-fucosyltransferase FUT5	AI-10-49	0.6705861
Q86W28	NACHT, LRR and PYD domains-containing protein 8	AI-10-49	0.6705852
O43566	Regulator of G-protein signaling 14	Q-203	0.67055416
Q9BVI0	PHD finger protein 20	Q-203	0.67055416
Q93074	Mediator of RNA polymerase II transcription subunit 12	PF-05190457	0.67054135
P51530	DNA replication ATP-dependent helicase/nuclease DNA2	PF-05190457	0.6705413
Q6NS38	DNA oxidative demethylase ALKBH2	AI-10-49	0.67052937
B4DJY2	Transmembrane protein 233	AI-10-49	0.67052513
Q8N807	Protein disulfide-isomerase-like protein of the testis	PF-05190457	0.67051905
P13929	Beta-enolase	AI-10-49	0.67051786
Q9NRJ7	Protocadherin beta-16	AI-10-49	0.67051774
O95751	Protein LDOC1	MK-5046	0.67051023

Table 2 continued from previous page

Uniprot	Protein name	Drug name	Probscore
Q92947	Glutaryl-CoA dehydrogenase, mitochondrial	fenebrutinib	0.67050344
Q96J94	Piwi-like protein 1	fenebrutinib	0.6704877
O15400	Syntaxin-7	adapalene	0.67046946
O00192	Armadillo repeat protein deleted in velo-cardio-facial syndrome	adapalene	0.67046934
Q17RD7	Synaptotagmin-16	CFI-402257	0.6704517
A0PJZ3	Glucoside xylosyltransferase 2	MK-5046	0.67042667
Q53GG5	PDZ and LIM domain protein 3	PF-05190457	0.6704057
P20929	Nebulin	PF-05190457	0.6704056
Q5NDL2	EGF domain-specific O-linked N-acetylglucosamine transferase	abemaciclib	0.67039376
Q10570	Cleavage and polyadenylation specificity factor subunit 1	fenebrutinib	0.6703786
Q53FE4	Uncharacterized protein C4orf17	AI-10-49	0.67036396
Q96T21	Selenocysteine insertion sequence-binding protein 2	cilofexor	0.67036384
P17980	26S proteasome regulatory subunit 6A	PF-05190457	0.67036086
Q8TE77	Protein phosphatase Slingshot homolog 3	tropifexor	0.67035466
Q96LD4	E3 ubiquitin-protein ligase TRIM47	tropifexor	0.67035466
P25205	DNA replication licensing factor MCM3	fenebrutinib	0.6703515
Q9NQC7	Ubiquitin carboxyl-terminal hydrolase CYLD	fenebrutinib	0.6703515
Q71F23	Centromere protein U	NMS-P715	0.6703202
P46778	60S ribosomal protein L21	fenebrutinib	0.67028475
Q04727	Transducin-like enhancer protein 4	AI-10-49	0.6702773
O14556	Glyceraldehyde-3-phosphate dehydrogenase, testis-specific	AI-10-49	0.6702773
Q92994	Transcription factor IIIB 90 kDa subunit	fenebrutinib	0.67027074
Q15646	2'-5'-oligoadenylate synthase-like protein	fenebrutinib	0.67027074
O15131	Importin subunit alpha-6	fenebrutinib	0.6702412
Q14254	Flotillin-2	fenebrutinib	0.6702412
O75764	Transcription elongation factor A protein 3	CFI-402257	0.67017365
P40425	Pre-B-cell leukemia transcription factor 2	MK-5046	0.67016166
O43766	Lipoyl synthase, mitochondrial	PF-05190457	0.67014426
Q969L2	Protein MAL2	fenebrutinib	0.67013794
Q9NSG2	Uncharacterized protein C1orf112	CCT137690	0.67013377
Q9H892	Tetratricopeptide repeat protein 12	PF-05190457	0.67012644
O95248	Myotubularin-related protein 5	CCT137690	0.6701216
O60481	Zinc finger protein ZIC 3	PF-05190457	0.670091
Q9UBK8	Methionine synthase reductase	AI-10-49	0.6700795
Q4G0J3	La-related protein 7	CCT137690	0.67007875
Q8WW32	High mobility group protein B4	CCT137690	0.67005986
P23297	Protein S100-A1	MK-5046	0.67002577
Q8TCB7	tRNA N	elbasvir	0.6700179
Q8IXH6	Tumor protein p53-inducible nuclear protein 2	abemaciclib	0.67000955

Table S 2: Undruggable human disease-associated proteins selected by ProtalCG

When we consider the proteins in Tbio, there are 9545 proteins which are not int Casas's druggable proteins. If 0.67 was used as the cutoff, 219 proteins were predicted as positive hits. The gene enrichment analysis result for these proteins was listed in Table S4. Disease associated with these 219 human proteins were also listed in Table S5. Since one protein is always related with multiple diseases, these diseases are ranked by the number of their associated proteins and the top 10 diseases were listed in the table. Most of top ranked diseases are related with cancer development. 21 drugs that are approved or in clinical trial are predicted to interact with these proteins as shown in Table S6.

If the proteins in Tbio were removed from the undruggable list, only 2930 proteins were left.

Drug name	Clinical phase	Mechanism of Action	Number of targeted proteins
AI-10-49	Preclinical	core binding factor inhibitor	63
Fenebrutinib	Phase 2	Bruton's tyrosine kinase (BTK) inhibitor	56
PF-05190457	Phase 2	growth hormone secretagogue receptor inverse agonist	42
Abemaciclib	Launched	CDK inhibitor	21
MK-5046	Preclinical	bombesin receptor agonist	18
CFI-402257	Phase 1/Phase 2	dual specificity protein kinase inhibitor	14
CCT137690	Preclinical	Aurora kinase inhibitor	11
Tropifexor	Phase 2	FXR agonist	5
NMS-1286937	Phase 2	PLK inhibitor	5
NMS-P715	Preclinical	protein kinase inhibitor	5
Elbasvir	Launched	HCV inhibitor	5
Cilofexor	Phase 3	FXR agonist	4
CDK9-IN-6	Preclinical	CDK inhibitor	3
piperaquine-phosphate	Launched	antimalarial agent	3
Q-203	Phase 2	ATP synthase inhibitor	3
ABT 702 dihydrochloride	Preclinical	adenosine kinase inhibitor	2
Ziritaxestat	Phase 3	autotaxin inhibitor	2
Adapalene	Launched	retinoid receptor agonist	1
Acalabrutinib	Launched	Bruton's tyrosine kinase (BTK) inhibitor	1
IACS-10759 Hydrochloride	Preclinical	mitochondrial complex I inhibitor	1
NVP-CGM097	Phase 1	MDM inhibitor	1

Table S 3: Chemicals interacted with undruggable human proteins

If 0.67 was used as the cutoff, there will be only 41 proteins predicted positive and no significant enrichment with David gene enrichment analysis. So 0.665 was used as a cutoff, and 348 proteins were predicted as positive hits. The gene enrichment analysis result for these proteins was listed in Table S7. Disease associated with these 348 human proteins were also listed in Table S8. 42 drugs that are approved or in clinical trial are predicted to interact with these proteins as shown in Table S9.

David Functional Annotation enrichment analysis				
Enriched terms in UniProtKB keywords	Number of proteins involved	Percentage of proteins involved	P-value	Modified Benjamini p-value
Alternative splicing	153	69.9	2.70E-08	6.80E-06
Phosphoprotein	126	57.5	1.70E-07	2.20E-05
Cytoplasm	82	37.4	3.20E-06	2.40E-04
Nucleus	87	39.7	3.70E-06	2.40E-04
Metal-binding	61	27.9	2.00E-04	1.00E-02

Table S 4: Functional Annotation enrichment for human proteins in Tbio selected by PortalCG

Disease Name	# of undruggable proteins associated with the disease
Breast Carcinoma	89
Tumor Cell Invasion	85
Carcinogenesis	83
Neoplasm Metastasis	73
Colorectal Carcinoma	68
Liver carcinoma	66
Non-Small Cell Lung Carcinoma	56
Malignant neoplasm of lung	56
Carcinoma of lung	54
Alzheimer's Disease	54

Table S 5: Top ranked diseases associated with proteins in Tbio selected by portal learning

rug name	Clinical phase	Mechanism of Action	Number of targeted proteins
AI-10-49	Preclinical	core binding factor inhibitor	52
fenebrutinib	Phase 2	Bruton's tyrosine kinase (BTK) inhibitor	45
PF-05190457	Phase 2	growth hormone secretagogue receptor inverse agonist	36
abemaciclib	Launched	CDK inhibitor	20
MK-5046	Preclinical	bombesin receptor agonist	15
CFI-402257	Phase 1/Phase 2	dual specificity protein kinase inhibitor	14
CCT137690	Preclinical	Aurora kinase inhibitor	7
NMS-1286937	Phase 2	PLK inhibitor	5
tropifexor	Phase 2	FXR agonist	4
NMS-P715	Preclinical	protein kinase inhibitor	4
cilofexor	Phase 3	FXR agonist	3
CDK9-IN-6	Preclinical	CDK inhibitor	3
piperaquine-phosphate	Launched	antimalarial agent	3
elbasvir	Launched	HCV inhibitor	3
ABT-702	Preclinical	adenosine kinase inhibitor	2
adapalene	Launched	retinoid receptor agonist	2
Q-203	Phase 2	ATP synthase inhibitor	2
ziritaxestat	Phase 3	autotaxin inhibitor	2
acalabrutinib	Launched	Bruton's tyrosine kinase (BTK) inhibitor	1
IACS-10759	Preclinical	mitochondrial complex I inhibitor	1
CGM097	Phase 1	MDM inhibitor	1

Table S 6: Chemicals interacted with human proteins in Tbio

David Functional Annotation enrichment analysis				
Enriched terms in UniProtKB keyword	Number of proteins involved	Percentage of proteins involved (%)	P-value	Modified Benjamini p-value
Zinc-finger	80	23	7.60E-16	1.20E-13
Zinc	85	24.4	1.20E-11	9.90E-10
Metal-binding	96	27.6	4.30E-06	2.30E-04
DNA-binding	62	17.8	7.90E-06	3.20E-04
Transcription regulation	61	17.5	5.60E-04	1.80E-02
Transcription	61	17.5	1.10E-03	3.00E-02

Table S 7: Functional Annotation enrichment for undruggable human disease proteins without Tbio selected by PortalCG

Disease Name	# of undruggable proteins associated with the disease
Body Height	31
Colorectal Carcinoma	28
Malignant neoplasm of breast	26
Breast Carcinoma	18
Blood Protein Measurement	18
Leukemia, Myelocytic, Acute	17
Carcinogenesis	17
Neoplasm Metastasis	17
Liver carcinoma	15
Malignant neoplasm of prostate	15

Table S 8: Top ranked diseases associated with undruggable human proteins excluding Tbio selected by portal learning

Drug_name	Clinical phase	Mechanism of Action	Number of targeted proteins
fenebrutinib	Phase 2	Bruton's tyrosine kinase (BTK) inhibitor	80
PF-05190457	Phase 2	growth hormone secretagogue receptor inverse agonist	50
MK-5046	Preclinical	bombesin receptor agonist	38
CCT137690	Preclinical	Aurora kinase inhibitor	36
AI-10-49	Preclinical	core binding factor inhibitor	31
abemaciclib	Launched	CDK inhibitor	26
CFI-402257	Phase 1/Phase 2	dual specificity protein kinase inhibitor	20
NMS-P715	Preclinical	protein kinase inhibitor	14
NMS-1286937	Phase 2	PLK inhibitor	11
elbasvir	Launched	HCV inhibitor	8
cilofexor	Phase 3	FXR agonist	7
ABBV-744	Phase 1	bromodomain inhibitor	7
tropifexor	Phase 2	FXR agonist	7
CDK9-IN-6	Preclinical	CDK inhibitor	6
adapalene	Launched	retinoid receptor agonist	4
Q-203	Phase 2	ATP synthase inhibitor	4
PLX8394	Phase 1/Phase 2	serine/threonine kinase inhibitor	4
ABT-702	Preclinical	adenosine kinase inhibitor	4
NVP-BSK805	Preclinical	JAK inhibitor	3
OTS167	Phase 1/Phase 2	maternal embryonic leucine zipper kinase inhibitor	3
CHIR-99021	Preclinical	glycogen synthase kinase inhibitor	3
DBPR-211	Preclinical	cannabinoid receptor antagonist	2
A-887826	Preclinical	sodium channel blocker	2
integrin-antagonist-1	Phase 1	integrin antagonist	2
piperaquine-phosphate	Launched	antimalarial agent	2
cenerimod	Phase 2	sphingosine 1-phosphate receptor modulator	1
peposertib	Phase 1/Phase 2	DNA dependent protein kinase inhibitor	1
tezacaftor	Launched	CFTR channel agonist	1
cot-inhibitor-2	Preclinical	MAPK-interacting kinase inhibitor	1
itacitinib	Phase 3	JAK inhibitor	1
10-hydroxycamptothecin	Preclinical	topoisomerase inhibitor	1
alectinib	Launched	ALK tyrosine kinase receptor inhibitor	1
adarotene	Phase 1	retinoid receptor agonist	1
acalabrutinib	Launched	Bruton's tyrosine kinase (BTK) inhibitor	1
XL041	Preclinical	LXR agonist	1
WAY-207024	Preclinical	gonadotropin releasing factor hormone receptor antagonist	1
MK-5108	Phase 1	Aurora kinase inhibitor	1
CGM097	Phase 1	MDM inhibitor	1
CD-437	Preclinical	retinoid receptor agonist	1
AMG-925	Phase 1	CDK inhibitor|FLT3 inhibitor	1
ACT-132577	Launched	endothelin receptor antagonist	1
ziritaxestat	Phase 3	autotaxin inhibitor	1

Table S 9: Chemicals interacted with undruggable human proteins excluding Tbio

Protein descriptor	layers	Albert –>Resnet
	embedding dimension	256
Chemical descriptor	backbone	GIN
	number of layers	5
	embedding dimension	300
	aggregation methods	sum
	drop out ratio	0.5
Interaction learner	layers	Attentive pooling –>2 layers of MLP
	embedding dimension	128
Structure residue-atom pair wise feature learner	layers	matrix multiplication of protein and chemical embedding vectors
Classifier	layers	2 layers of MLP
	embedding dimension	64

Table S 10: Model architecture configuration

4 Related works

4.1 OOD generalization in deep learning

The recent work Invariant risk minimization (IRM)[7] is a dedicated algorithm to OOD generalization, which is under the goal of transformative solution for invariant representation. However, given its completeness in theory, many experiments [23] report IRM are not doing well in large real-world data set.

Many deep learning tasks are inherently OOD generalization. Among those jargon, some are famous for defining a type of OOD scenario problem, for example, Domain generalization [24] can be taken as the equivalent of OOD generalization; domain shift [24] rephrases the fact of distribution change in terms of $D(X, y)$. Some jargon define a type of solution: domain alignment [25] minimizes the difference/distance between source domains and target domains distributions for an invariant representation where the distance between source and target domain distributions are measured by a wide variety of statistical distance metrics from simple l_2, $f - divergence$ to Wasserstein distance; domain adaptation [26] is to leverage pretrained model on a different domain and is just one idea to achieve domain generalization, the more general term that is equivalent to OOD generalization in a more practical sense; causal learning is proved by [6] to be equivalent to OOD generalization when causality makes senses (taking into consideration the existence of cases where causality is meaningless); robust optimization [27] that focuses on worst-group performance instead of the average one in ERM; although robust optimization has not quite been adapted to modern deep learning, its sub-field distributional robust optimization [28] has witnessed quite a few recent works adapted to be used in deep learning.

Worth to be clarified that, many works that are solving the sub-group or sub-population shift problem is quite different from the OOD generalization problem as discussed in the setting of dark chemical genomics space. Sub-population shift is more like a imbalanced data problem where the test set has major resemblance with training data just the shift from a major class to a minor class or vise-versa. For example, GroupDRO [29] was published in 2018 to address this problem, proposing to incorporate structural assumptions on distribution, which could be straight forward in some data sets which has more meta-data or is a multi-label classification case where the label structure could be used as the structural assumption.

4.2 Portal learning key components related

(Model architecture) Even since the debut of the survey [30] enlightening the perspective of *representation learning*, enormous research passion is motivated for model architecture design, almost taken as equivalent to deep learning and overshadowing all other directions. A key idea that echos the demand of generalization is to learn *global representation* which helps to decrease both $TrainError(f_i^{iid})$ and *known space generalization gap*, denoising large data set. Hence, to solve OOD, good model architecture design is not enough.

All existing work in CPI is confined in the known space and limited works have concerned generalization. Generally, proposed CPI deep learning models follow the same fashion: build model architecture of three key modules, protein descriptor, chemical descriptor and interaction learner formulating a classification problem with a few variants as regression problem. Innovation is seen mostly for model architecture, particularly active for chemical descriptor, reflecting all milestones in recent years deep learning advancement from CNN, LSTM to Transformer and GNN as demonstrated in DeepPurpose [31]. Generalization has not shown in any previous work as a main goal of research except for DISAE[14] which proves generalizability to orphan GPCR protein drug screening mainly relying on a general purpose pretrained protein language model. It's fine-tuned on GPCR data set with shifted evaluation. Hence, DISAE becomes the baseline model in this work.

(Model intialization) Although could be categorized as a type of representation learning, *transfer learning* became an iconic independent concept for its huge success with breakthroughs in many NLP and CV benchmark tasks. It features a *pretraining-finetuning* procedure. An intuitive example is to pretrain a language model on large general English vocabulary with pretext task formulation such as predicting next word and then to finetune the language model on specific downstreawm task such as machine translation in biology domain. Well-renowned Transformer based pretrained models starting from human language models are a combined success of attention based model architecture design and transfer learning. In the computation biology field, most eye-catching equivalent is protein language model, i.e. protein descripto, which inspired several similar works at the same time by different groups: TAPE and ESM showcases pretraining on large protein vocabulary could significantly improves downstream task such as protein-protein interaction prediction; MSA-based-tranformer and DISAE incorporates MSA in pretraining. From the perspective of the target downstrem task, the power of transfer learning comes from a better model initialization. This is a major breakthrough that could fill the gap of $TestError(f_t^{OOD}) - TestError(f_t^{iid})$ but not

necessarily, depending on how it's incorporated into the whole training scheme at system level, particularly depending on data fed in.

DISAE is used in our work here as a pretrained protein descriptor. This choice over other protein language models is due to the fact that DISAE is the smallest among other in terms of memory required to use and optimize with same level of performance. STL is a way to leverage transfer learning to fine better model initialization. The main difference and innovation is that transfer learning naively relies on the belief that more general knowledge transferred will bring better performance while STL in portal learning actively leverage biology endorsed biased when transferring general knowledge. Further, by defining the goal "to learn the portal", which will be closer to global optimum in target universe loss landscape, the whole training system is steered actively solve ODD.

(STL) Sparked by the breakthrough of Alphafold 1 [17] and Alphafold 2[32] in protein structure prediction, deep learning has been trusted in molecule interaction distance map prediction to learn structure information. The inclusion of CPI-structure, i.e. protein function prediction portal calibration is inspired by recent success in protein structure prediction led by the great work of AlphaFold1[17] and AlphaFold2[32]. Specifically, we pretrain the model to predict residue-residue contacts for a protein whose structure is solved and chemical atom-protein residue contacts given a known CPI complex structure. There are three popular ways of residue-residue pairwise distance matrix prediction depending on how to formulate it as a machine learning task. On the one end is to formulate it as a binary classification where a distance threshold is set defining whether a pair of residues are in contact or not, hence the name contact prediction. On the other end is to formulate it as a regression problem where the exact distance is used as a regression target, hence the name exact distance prediction. AlphaFold1 showed another way in between the two ends, which is to formulate it as a multi-class classification problem, where the distribution of pair-wise residue distances is broken down into multiple class labels according to a histogram, hence the name distogram prediction. We first focus on residue-atom pair wise distance at binding sites and then experiments contact prediction and distogram prediction. In our results, the two formulations have similar performance in light of the final CPI prediction through ablation study as shown in Supplemental Figure S12.

(OOC-ML) It's long be aware that the sequence order of training data exposed to the model has an impact on model generalizability. Active learning [33] emphasises to actively query data in a iterative fashion to only expose the model to data close to he decision boundary . Curriculum learning [34] emphasises to sort all training so that the model is exposed to challenges of increasing difficulty. This element of data logistics has also been closely weaved into many optimization algorithms that aim to improve model generalizability. For example, contrastive loss[35] requires certain ratio of positive v.s. negative samples in each mini-bath. Most related to portal learning is meta-learning which can be categorized into metric-based, model-based and optimizer based "learn to learn" algorithms [36] with application to few-shot learning and zero-shot learning. Meta-learning started for the data-efficiency challenge instead of generalization or OOD. Although meta-learning is defined very general, making many algorithms seem to be mere an variants falling under its umbrella, in practice, algorithms proposed bearing the name of meta-learning are defined on multi-class classification data set, typically image classification, where the main challenge is the huge number of classes while limited data points are known in each class. Because of this underlying motivation, meta-learning features a very involving data logistics with multiple layer of optimization each has its meta-train/meta-test set sampled based on label distribution. These unspecified facts reveal that there is no existing meta-learning algorithm fit into CPI data.

However, the idea of "learn to learn" is attractive. MAML[21] is the optimization based meta-learning work that inspired OOC-ML proposed as a major component of portal learning. The differences are major. OOC-ML algorithm expands on it by focusing on data feature distribution instead of label distribution, encouraging active sampling in local neighborhood, which simplifies the support/query meta-train/meta-test data logistics, and ensembling a few local loss directions to learn global gravity direction.

5 Additional figures

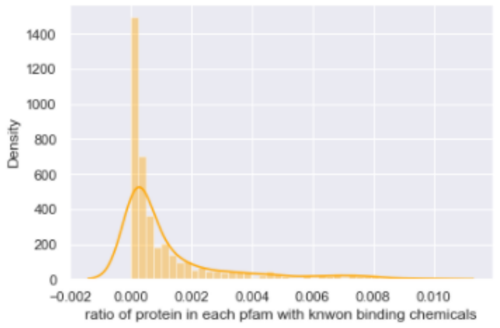

Figure S 1: Dark space statistics histogram based on known CPI pairs in ChEMBL26. < 1% proteins in each pfam invovled in ChEMBLE have known binding chemcials.

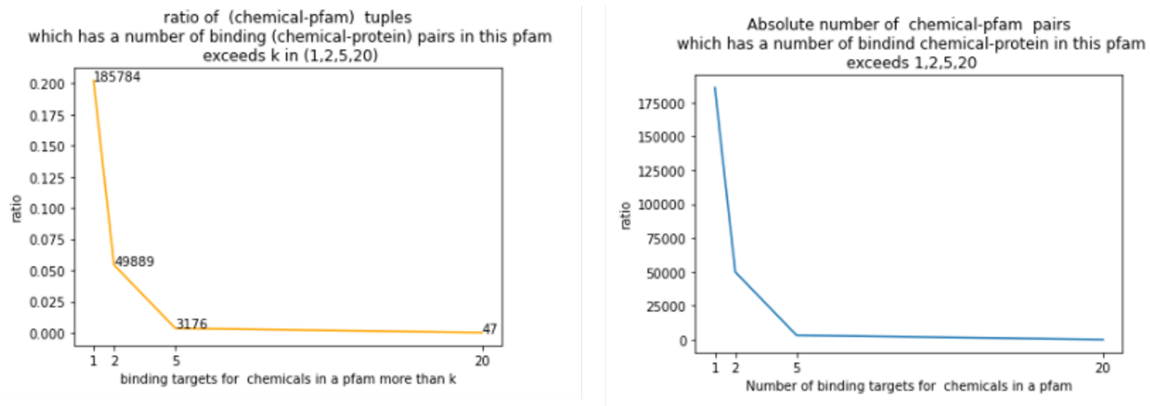

Figure S 2: Dark space statistics histogram based on known CPI pairs in ChEMBL26. < 1% chemicals bind to more then 2 proteins; < 0.4% chemicals bind to more than 5 proteins.

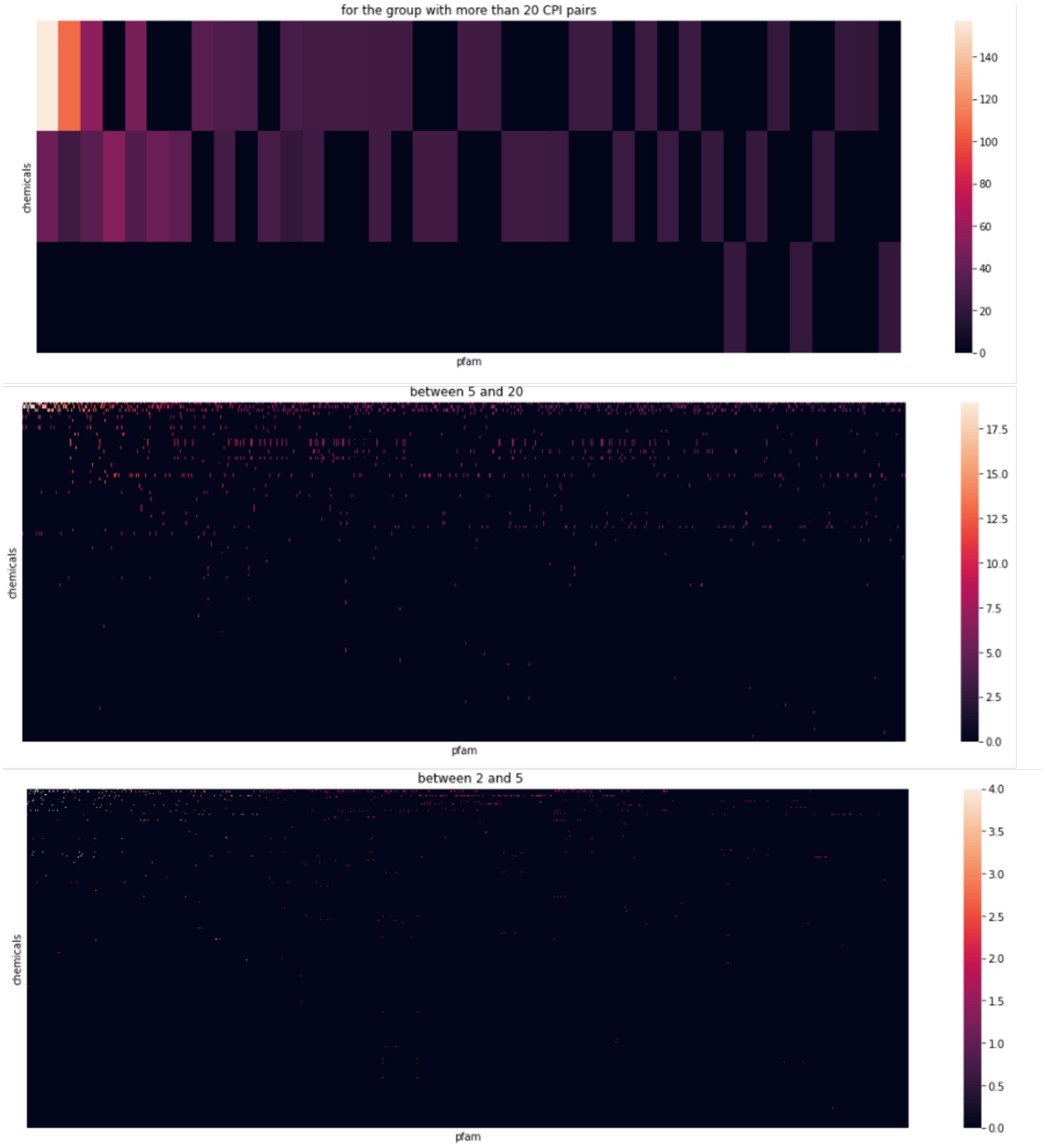

Figure S 3: As shown in Figure S 2, there are three main ranges in terms of the binding targets in a pfam for one chemical: [2,5],[5,20],[20,). For each of the range, a heatmap is shown with y axis representing each chemicals, x axis representing each pfam, each point representing the known binding pairs for one chemical and one pfam. As we can see, there is huge dark space.

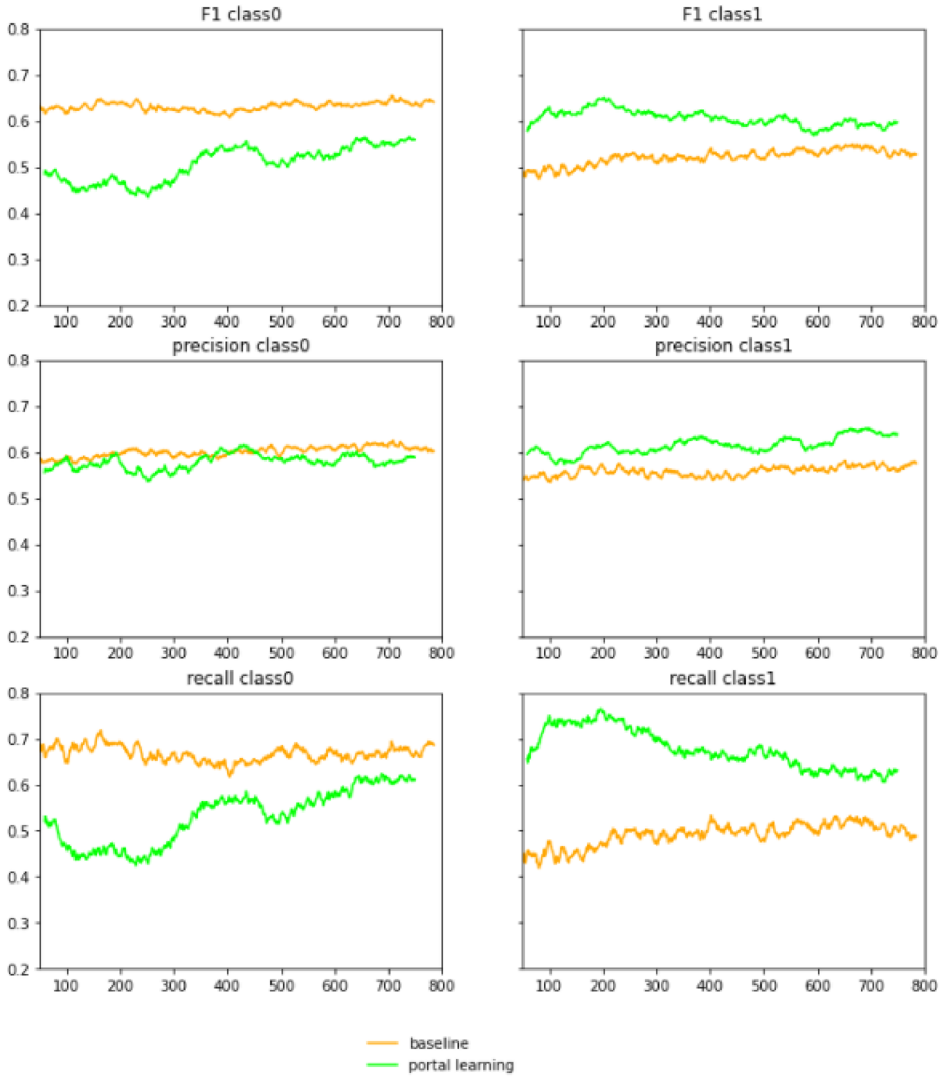

Figure S 4: In the main text, overall evaluation across positive and negative classes are reported, such as F1, ROC-AUC, PR-AUC. Here is a breakdown of performance in each class, where class0 is negative, i.e. not binding, class1 is positive, i.e. binding. against DISAE as baseline

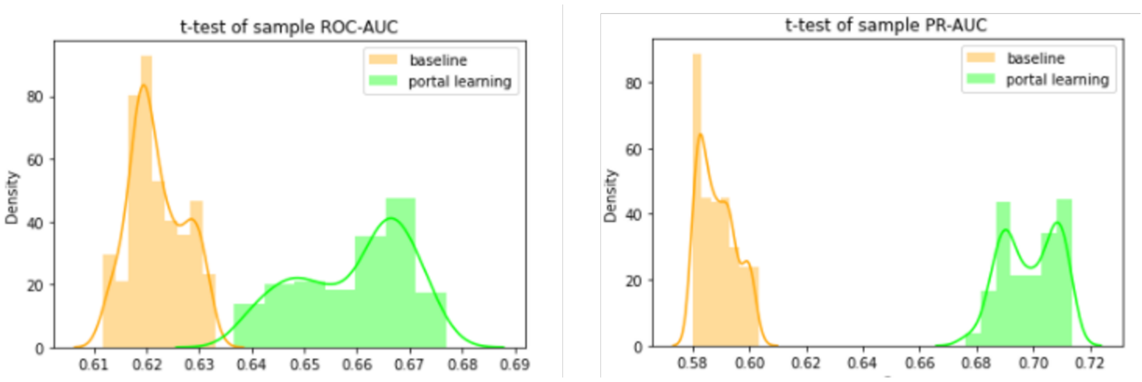

Figure S 5: t-test comparison. The p-values for both ROC-AUC and PR-AUC are close to 0 against DISAE as baseline

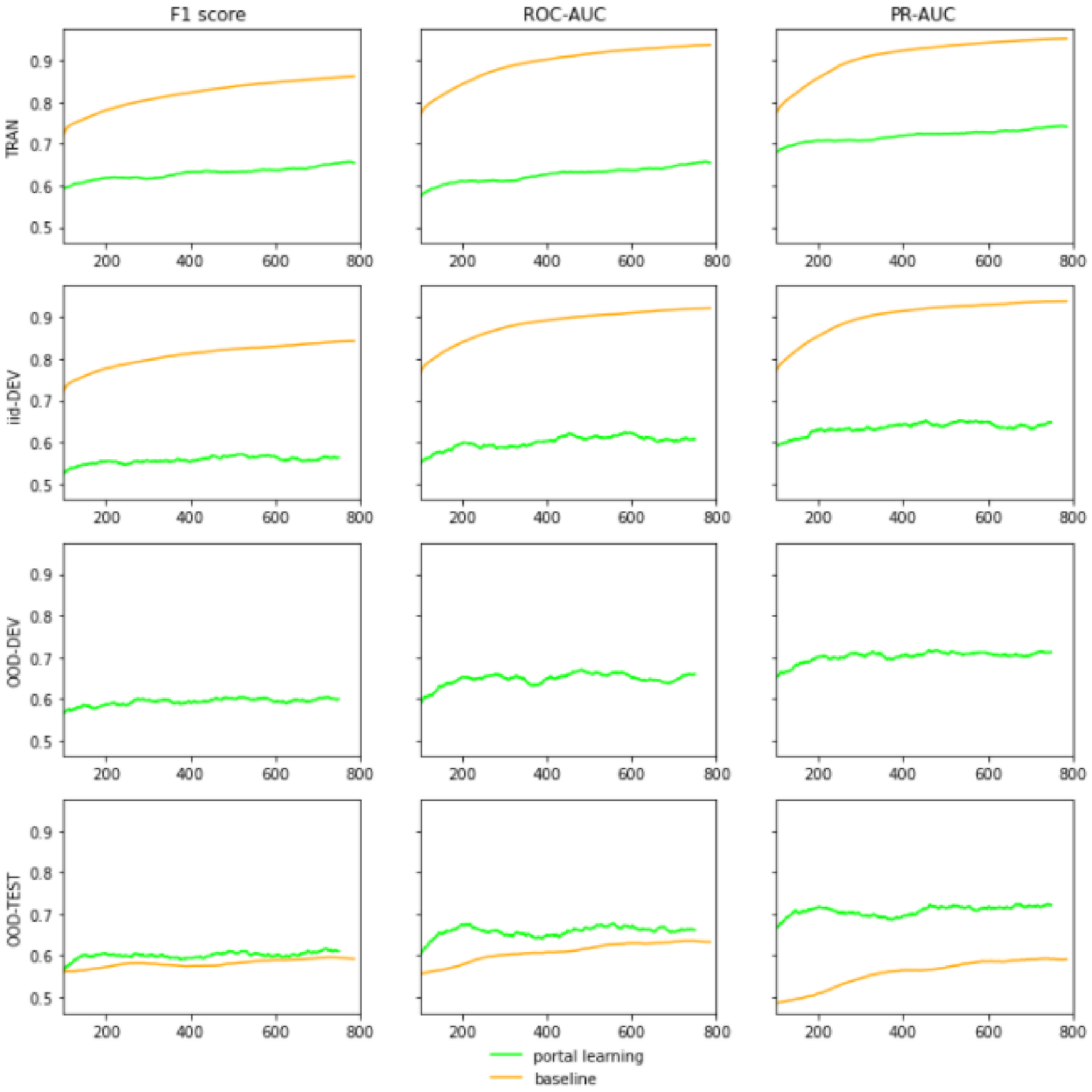

Figure S 6: Stress model selection performance curves against DISAE as baseline

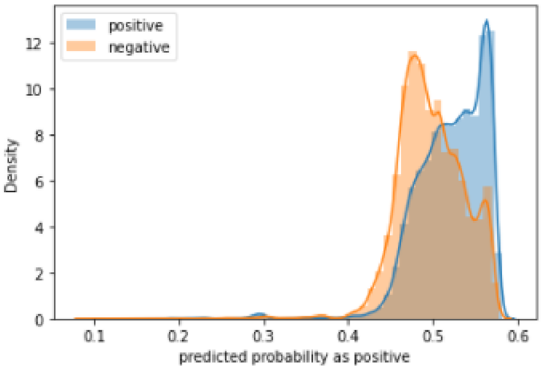

Figure S 7: To select high confidence prediction, one additional procedure of filtering is to build a histogram of prediction scores based on known pairs. A threshold of 0.67 is identified to filter out confident positive prediction.

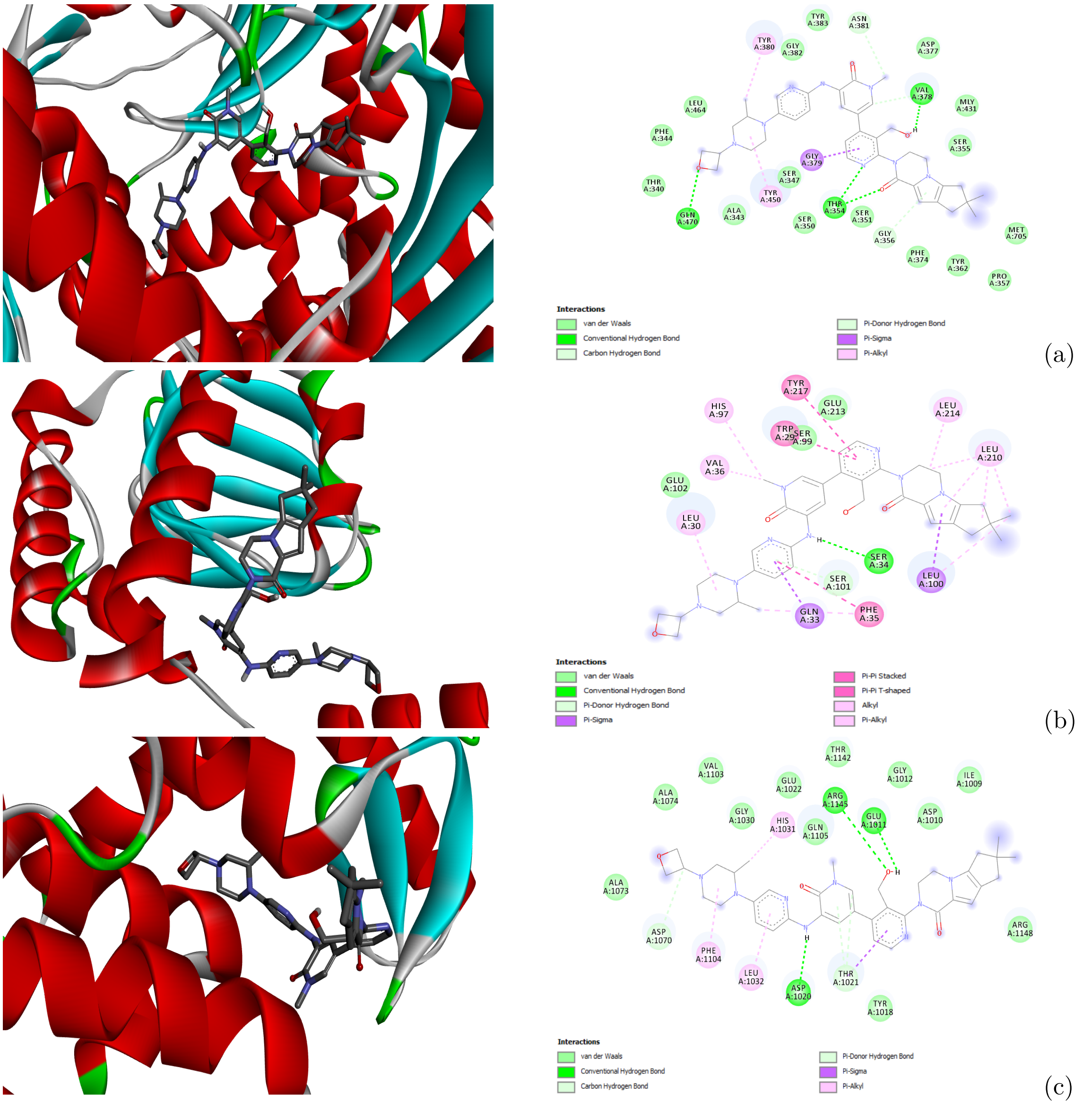

Figure S 8: The binding conformations and 2D-interactions of Fenebrutinib on the targeted proteins predicted by using Autodock: (a) Fenebrutinib on Presequence protease. (b) Fenebrutinib on Sigma non-opioid intracellular receptor 1 . (c) Fenebrutinib on Mitochondrial amidoxime-reducing component 1.

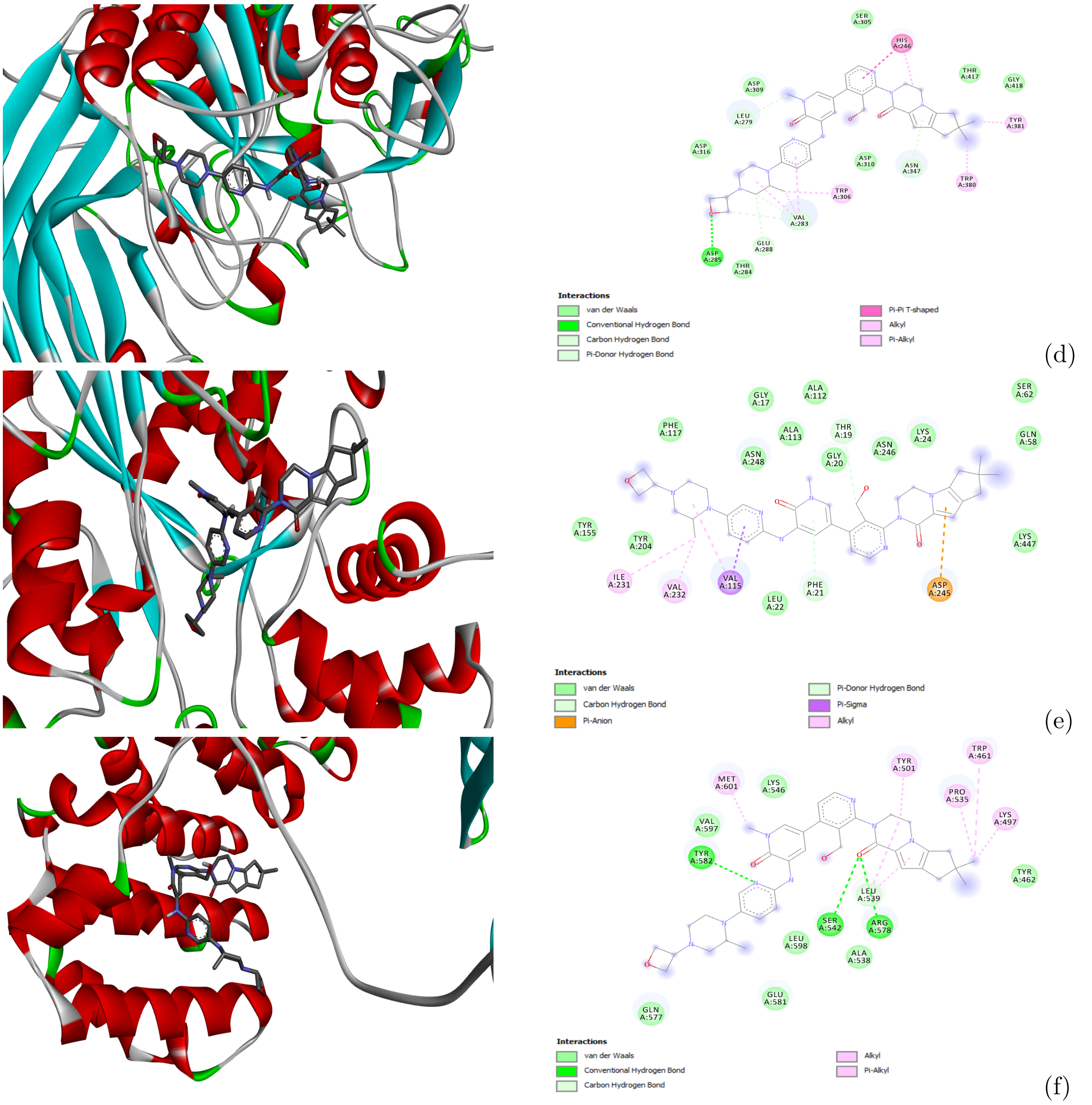

Figure S 9: The binding conformations and 2D-interactions of Fenebrutinib on the targeted proteins predicted by using Autodock: (d) Fenebrutinib on Proprotein convertase subtilisin/kexin type 6. (e) Fenebrutinib on Fatty acyl-CoA reductase 2. (f) Fenebrutinib on AP-2 complex subunit alpha-2.

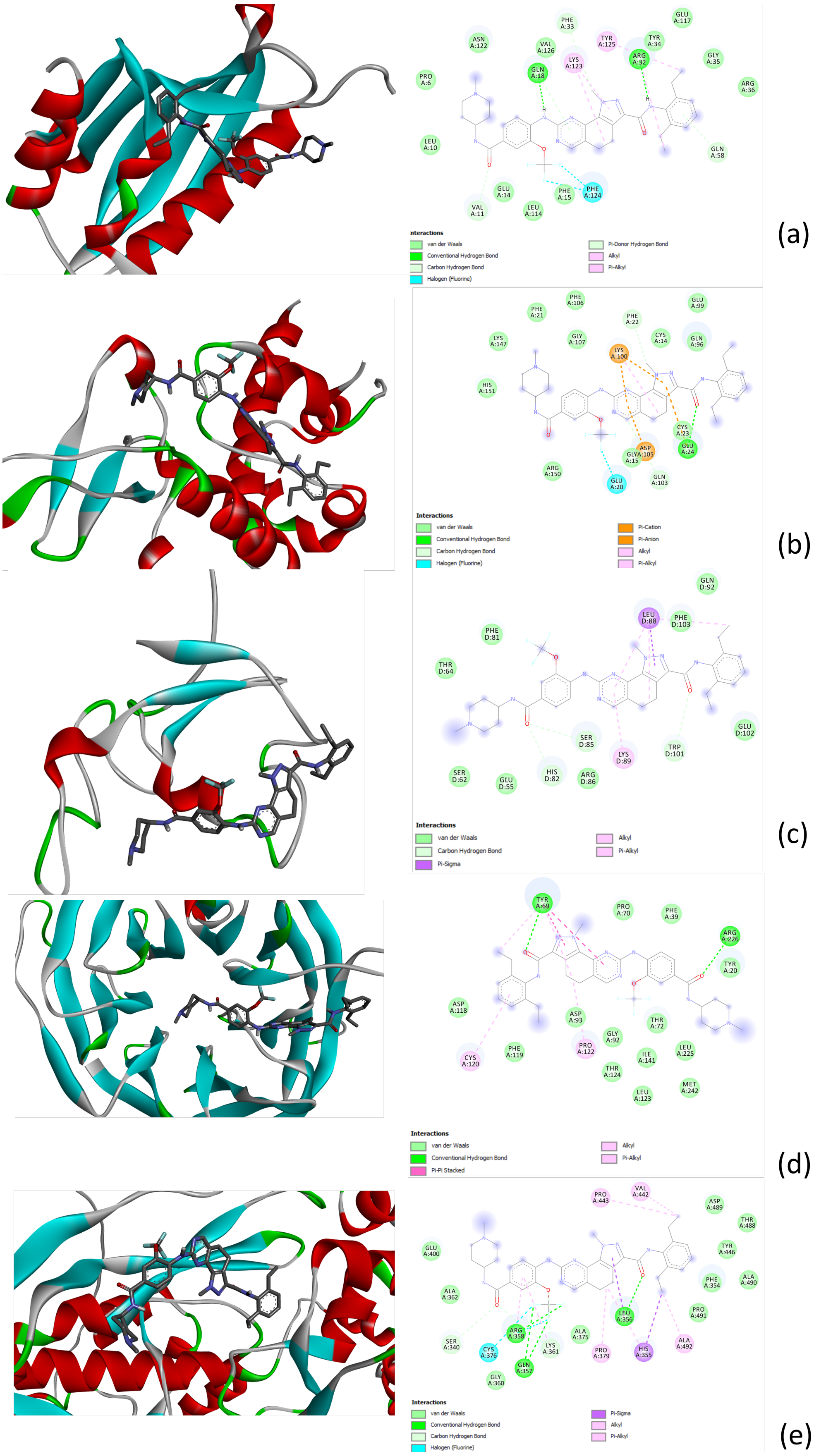

Figure S 10: The binding conformations and 2D-interactions of NMS-P715 on the targeted proteins predicted by using Autodock: (a) NMS-P715 on Ras GTPase-activating protein-binding protein 2. (b) NMS-P715 on Casein kinase II subunit beta. (c) NMS-P715 on E3 ubiquitin-protein ligase RBX1. (d) NMS-P715 on DDB1- and CUL4-associated factor 7.(e) NMS-P715 on tRNA (guanine(26)-N(2))-dimethyltransferase.

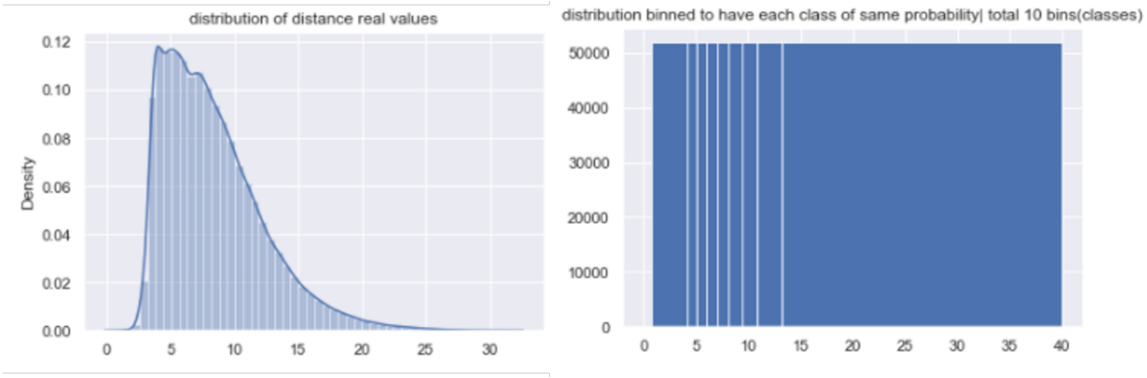

Figure S 11: Histogram equalization results: the left panel shows the original distribution of distance real values; to formalize a multi-class classification where each class has equal probability, histogram equalization transforms the distribution to the right panel of 10 bins, each as a class.

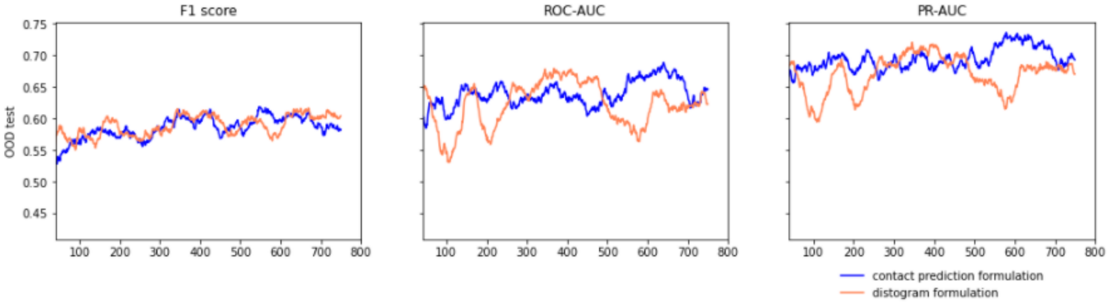

Figure S 12: Ablation study to compare the impact of two formulation of protein structure distance prediction, contact prediction v.s. distogram prediction. The two variants have similar OOD-test performance.

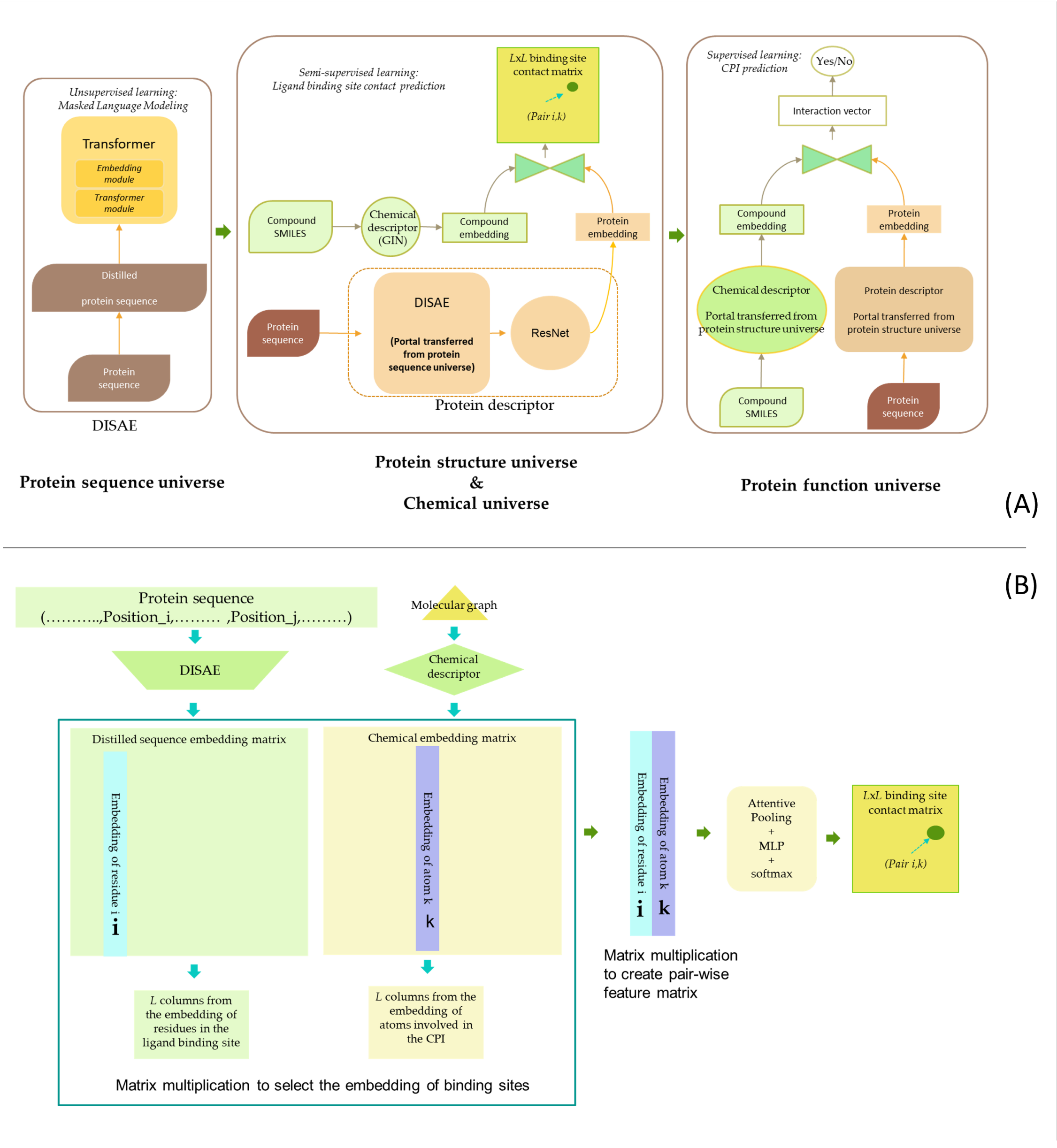

Figure S 13: Illustration of PortalCG architecture: (A) the pipeline of STL (B) The model architecture for predicting binding site distance matrix. Note that Portal Learning is a general framework at the training scheme level instead of at the model architecture level. OOC-ML as an optimization algorithm is only used in the protein function universe which is not a model architecture component (See Figure 1).

References

[1] I. Goodfellow, Y. Bengio, and A. Courville, *Deep Learning.* MIT Press, 2016. `http://www.deeplearningbook.org`.

[2] H. Li, Z. Xu, G. Taylor, C. Studer, and T. Goldstein, "Visualizing the loss landscape of neural nets," *arXiv preprint arXiv:1712.09913*, 2017.

[3] P. Nakkiran, B. Neyshabur, and H. Sedghi, "The deep bootstrap: Good online learners are good offline generalizers," *arXiv preprint arXiv:2010.08127*, 2020.

[4] Z. Lan, M. Chen, S. Goodman, K. Gimpel, P. Sharma, and R. Soricut, "Albert: A lite bert for self-supervised learning of language representations," *arXiv preprint arXiv:1909.11942*, 2019.

[5] J.-Y. Le Boudec, *Performance evaluation of computer and communication systems*, vol. 2. Epfl Press Lausanne, 2010.

[6] M. Arjovsky, "Out of distribution generalization in machine learning," 2021.

[7] M. Arjovsky, L. Bottou, I. Gulrajani, and D. Lopez-Paz, "Invariant risk minimization," 2020.

[8] A. D'Amour, K. Heller, D. Moldovan, B. Adlam, B. Alipanahi, A. Beutel, C. Chen, J. Deaton, J. Eisenstein, M. D. Hoffman, *et al.*, "Underspecification presents challenges for credibility in modern machine learning," *arXiv preprint arXiv:2011.03395*, 2020.

[9] V. Vapnik, "Principles of risk minimization for learning theory," in *Advances in neural information processing systems*, pp. 831–838, 1992.

[10] J. Mistry, S. Chuguransky, L. Williams, M. Qureshi, G. A. Salazar, E. L. Sonnhammer, S. C. Tosatto, L. Paladin, S. Raj, L. J. Richardson, *et al.*, "Pfam: The protein families database in 2021," *Nucleic Acids Research*, vol. 49, no. D1, pp. D412–D419, 2021.

[11] H. M. Berman, J. Westbrook, Z. Feng, G. Gilliland, T. N. Bhat, H. Weissig, I. N. Shindyalov, and P. E. Bourne, "The Protein Data Bank," *Nucleic Acids Research*, vol. 28, pp. 235–242, 01 2000.

[12] J. Yang, A. Roy, and Y. Zhang, "Biolip: a semi-manually curated database for biologically relevant ligand–protein interactions," *Nucleic acids research*, vol. 41, no. D1, pp. D1096–D1103, 2012.

[13] A. Gaulton, A. Hersey, M. Nowotka, A. P. Bento, J. Chambers, D. Mendez, P. Mutowo, F. Atkinson, L. J. Bellis, E. Cibrián-Uhalte, M. Davies, N. Dedman, A. Karlsson, M. P. Magariños, J. P. Overington, G. Papadatos, I. Smit, and A. R. Leach, "The ChEMBL database in 2017," *Nucleic Acids Research*, vol. 45, pp. D945–D954, 11 2016.

[14] T. Cai, H. Lim, K. A. Abbu, Y. Qiu, R. Nussinov, and L. Xie, "Msa-regularized protein sequence transformer toward predicting genome-wide chemical-protein interactions: Application to gpcrome deorphanization," *Journal of Chemical Information and Modeling*, vol. 61, no. 4, pp. 1570–1582, 2021.

[15] S. C. Potter, A. Luciani, S. R. Eddy, Y. Park, R. Lopez, and R. D. Finn, "Hmmer web server: 2018 update," *Nucleic acids research*, vol. 46, no. W1, pp. W200–W204, 2018.

[16] K. Xu, W. Hu, J. Leskovec, and S. Jegelka, "How powerful are graph neural networks?," *arXiv preprint arXiv:1810.00826*, 2018.

[17] N. Hiranuma, H. Park, M. Baek, I. Anishchenko, J. Dauparas, and D. Baker, "Improved protein structure refinement guided by deep learning based accuracy estimation," *Nature communications*, vol. 12, no. 1, pp. 1–11, 2021.

[18] S. Boyd and L. Vandenberghe, *Introduction to applied linear algebra: vectors, matrices, and least squares.* Cambridge university press, 2018.

[19] C. d. Santos, M. Tan, B. Xiang, and B. Zhou, "Attentive pooling networks," *arXiv preprint arXiv:1602.03609*, 2016.

[20] K. He, X. Zhang, S. Ren, and J. Sun, "Deep residual learning for image recognition," in *Proceedings of the IEEE conference on computer vision and pattern recognition*, pp. 770–778, 2016.

[21] C. Finn, P. Abbeel, and S. Levine, “Model-agnostic meta-learning for fast adaptation of deep networks,” *CoRR*, vol. abs/1703.03400, 2017.

[22] O. Trott and A. J. Olson, “Autodock vina: improving the speed and accuracy of docking with a new scoring function, efficient optimization and multithreading,” *Journal of Computational Chemistry*, vol. 31, pp. 455–461, 2010.

[23] E. Rosenfeld, P. Ravikumar, and A. Risteski, “The risks of invariant risk minimization,” *arXiv preprint arXiv:2010.05761*, 2020.

[24] K. Zhou, Z. Liu, Y. Qiao, T. Xiang, and C. C. Loy, “Domain generalization: A survey,” *arXiv preprint arXiv:2103.02503*, 2021.

[25] K. Muandet, D. Balduzzi, and B. Schölkopf, “Domain generalization via invariant feature representation,” in *International Conference on Machine Learning*, pp. 10–18, PMLR, 2013.

[26] B. Kulis, K. Saenko, and T. Darrell, “What you saw is not what you get: Domain adaptation using asymmetric kernel transforms,” in *CVPR 2011*, pp. 1785–1792, IEEE, 2011.

[27] A. Ben-Tal, L. El Ghaoui, and A. Nemirovski, *Robust optimization*. Princeton university press, 2009.

[28] H. Rahimian and S. Mehrotra, “Distributionally robust optimization: A review,” 2019.

[29] W. Hu, G. Niu, I. Sato, and M. Sugiyama, “Does distributionally robust supervised learning give robust classifiers?,” in *International Conference on Machine Learning*, pp. 2029–2037, PMLR, 2018.

[30] Y. Bengio, A. C. Courville, and P. Vincent, “Unsupervised feature learning and deep learning: A review and new perspectives,” *CoRR*, vol. abs/1206.5538, 2012.

[31] K. Huang, T. Fu, L. M. Glass, M. Zitnik, C. Xiao, and J. Sun, “Deeppurpose: A deep learning library for drug-target interaction prediction,” *Bioinformatics*, 2020.

[32] J. Jumper, R. Evans, A. Pritzel, T. Green, M. Figurnov, O. Ronneberger, K. Tunyasuvunakool, R. Bates, A. Žídek, A. Potapenko, *et al.*, “Highly accurate protein structure prediction with alphafold,” *Nature*, pp. 1–11, 2021.

[33] M. Eisenstein, “Active machine learning helps drug hunters tackle biology.,” *Nature biotechnology*, vol. 38, no. 5, pp. 512–515, 2020.

[34] Y. Bengio, J. Louradour, R. Collobert, and J. Weston, “Curriculum learning,” in *Proceedings of the 26th annual international conference on machine learning*, pp. 41–48, 2009.

[35] G. Koch, R. Zemel, and R. Salakhutdinov, “Siamese neural networks for one-shot image recognition,” in *ICML deep learning workshop*, vol. 2, Lille, 2015.

[36] T. M. Hospedales, A. Antoniou, P. Micaelli, and A. J. Storkey, “Meta-learning in neural networks: A survey,” *CoRR*, vol. abs/2004.05439, 2020.
